# Hexokinase-2 depletion inhibits glycolysis and induces oxidative phosphorylation in hepatocellular carcinoma and sensitizes to metformin

**DOI:** 10.1038/s41467-017-02733-4

**Published:** 2018-01-31

**Authors:** Dannielle DeWaal, Veronique Nogueira, Alexander R. Terry, Krushna C. Patra, Sang-Min Jeon, Grace Guzman, Jennifer Au, Christopher P. Long, Maciek R. Antoniewicz, Nissim Hay

**Affiliations:** 1 Department of Biochemistry and Molecular Genetics, College of Medicine, University of Illinois at Chicago, Chicago, IL 60607 USA; 2 Research & Development Section, Jesse Brown VA Medical Center, Chicago, IL 60612 USA; 3 Department of Pathology, College of Medicine, Cancer Center, University of Illinois Hospital and Health Science Chicago, Chicago, IL 60612 USA; 4 Department of Chemical and Biomolecular Engineering, Metabolic Engineering and Systems Biology Laboratory, University of Delaware, Newark, DE 19716 USA; 5 Present Address: Massachusetts General Hospital Cancer Center, Harvard Medical School, Boston, MA 02114 USA; 6 Present Address: College of Pharmacy, Ajou University Yeongtong-gu, Suwon-si Gyeonggi-do, 443-749 Korea

## Abstract

Hepatocellular carcinoma (HCC) cells are metabolically distinct from normal hepatocytes by expressing the high-affinity hexokinase (HK2) and suppressing glucokinase (GCK). This is exploited to selectively target HCC. Hepatic HK2 deletion inhibits tumor incidence in a mouse model of hepatocarcinogenesis. Silencing HK2 in human HCC cells inhibits tumorigenesis and increases cell death, which cannot be restored by GCK or mitochondrial binding deficient HK2. Upon HK2 silencing, glucose flux to pyruvate and lactate is inhibited, but TCA fluxes are maintained. Serine uptake and glycine secretion are elevated suggesting increased requirement for one-carbon contribution. Consistently, vulnerability to serine depletion increases. The decrease in glycolysis is coupled to elevated oxidative phosphorylation, which is diminished by metformin, further increasing cell death and inhibiting tumor growth. Neither HK2 silencing nor metformin alone inhibits mTORC1, but their combination inhibits mTORC1 in an AMPK-independent and REDD1-dependent mechanism. Finally, HK2 silencing synergizes with sorafenib to inhibit tumor growth.

## Abstract (web summary)

Hexokinase 2 (HK2) is selectively upregulated in hepatocellular carcinoma (HCC). Here the authors show that HK2 ablation decreases glycolysis and triggers oxidative phosphorylation (OXPHO) rendering HCC more susceptible to the OXPHO inhibitor metformin and to the FDA-approved drug sorafenib.

## Introduction

HCC is the third deadliest cancer with over 600,000 deaths per year worldwide, but it is only the sixth most common cancer, indicating a lack of effective treatment options^1, 2^. Currently, the pan-kinase inhibitor sorafenib is the only FDA-approved drug for the treatment of HCC; thus, development of more effective therapeutic strategies is highly desirable. HCC cells are metabolically distinct from normal hepatocytes and express different metabolic enzymes^3^. Targeting an enzyme that is present only in HCC and not in the corresponding normal liver tissue could be used to selectively target HCC cells. Hexokinase 2 (HK2) represents one such target. Hexokinases catalyze the first committed step in glucose metabolism by phosphorylating glucose. There are five known hexokinase isoforms encoded by separate genes in mammalian cells^3^. HK1 is expressed most ubiquitously in adult tissues and is considered the housekeeping isoform, while HK2 is a more regulated form expressed in few adult tissues, including skeletal and cardiac muscle and adipose tissues^4^, but it is highly expressed in many fetal tissues and in cancer cells. HK3 is the least characterized because it is expressed at low levels in almost all tissues and is thought to be substrate-inhibited by physiologic concentrations of glucose. HK4, or glucokinase (GCK), is expressed primarily in the liver and pancreas^5^. HK1-3 are high-affinity hexokinases with low Km, whereas GCK is a low affinity hexokinase with a high Km. Hexokinases share high-sequence homology but differ in their kinetics, subcellular distribution, and regulation suited to their specific metabolic functions that are still not completely understood^5^. A fifth hexokinase was recently discovered but has not yet been fully characterized^6^. Both HK1 and HK2 bind to the outer mitochondrial membrane and voltage-dependent anion channel (VDAC), and are allosterically inhibited and released from mitochondria by their own catalytic product glucose-6-phosphate (G6P)^5^.

In normal differentiated hepatocytes, GCK is the major hexokinase (HK) isoform expressed; in HCC, GCK expression is repressed and expression of the fetal HK isoform, HK2, is induced^7^. Thus, in HCC cells, the predominantly expressed HK isoform is HK2; this distinguishes HCC cells from the normal surrounding adult hepatocytes. In a tumor tissue microarray (TMA) analysis of 312 samples from 153 human patients, we found that HK2 upregulation occurs at the onset of cirrhosis, increases in dysplasia, and is expressed to the greatest extent in carcinoma, suggesting that the level of HK2 correlates with hepatic disease progression regardless of cause^8^. Since HK2 is not expressed in most adult tissues, including adult hepatocytes, but is highly expressed in HCC, targeting HK2 may allow for the selective eradication of HCC with a greatly reduced potential for side effects. This was demonstrated by the systemic deletion of HK2 in adult mice with an absence of overt side effects^9^. Thus, HK2 could represent an ideal cancer-specific target for HCC therapy. To understand the role of HK2 in HCC, we deleted HK2 in a mouse model of hepatocarcinogenesis and silenced it in human HCC cell lines. We found that HK2 ablation inhibits hepatocarcinogenesis, proliferation and survival and in vivo tumor growth of HCC cells. HK2 ablation markedly inhibited glucose flux, but glutamine flux and the TCA cycle were maintained. Oxidative phosphorylation (OXPHO) was elevated as a consequence of HK2 ablation. The complex I inhibitor metformin inhibited the increase in OXPHO, and the combination of HK2 ablation and metformin were synergistic in increasing cell death and in inhibiting tumor growth in vivo. Metformin also synergized with HK2 deficiency to inhibit mTORC1 in an AMPK-independent and REDD1-dependent manner. Finally, HK2 deficiency markedly increased the susceptibility to cell death induced by the FDA-approved drug sorafenib and markedly increased sorafenib inhibition of tumor growth in vivo.

## Results

### HK2 expression is required for hepatocarcinogenesis

**Fig. 1:**
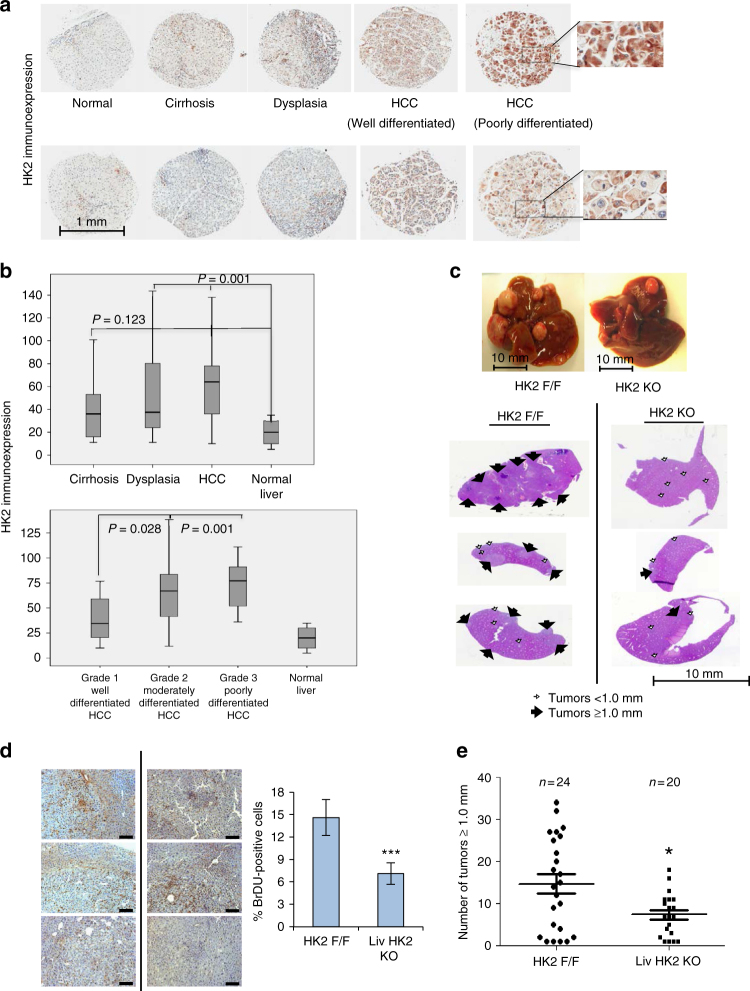
HK2 in the development of HCC. **a** Immunocytochemistry (IHC) for HK2 in human patient samples at various stages of liver disease (inserts show x3 magnfication). **b** Upper panel- quantification of HK2 expression in cirrhosis dysplasia and HCC. Significant differences for HCC (*p* < 0.001) and dysplasia (*p* = 0.001) vs. controls. No significant difference between cirrhosis and controls (*p* = 0.123 by Friedman test, cirrhosis (*n* = 106), dysplasia (*n* = 143), HCC (*n* = 45), and normal (*n* = 6)). Lower panel—quantification of HK2 expression within different grades of HCC. Poorly differentiated vs. well differentiated, *p* = 0.001. Moderately differentiated vs. well differentiated, *p* = 0.028 by ANOVA. Quantification is based on HK2 expression analysis of 312 samples from 151 patients exhibiting liver disease with different etiologies. For both panels box plot center line represents mean, box limits represent 25% and 75% confidence limits, and whiskers extend to the minimum and maximum values. **c** Two-week-old *HK2*^*F/F*^ and *HK2*^*F/F*^*;AlbCre* male mice were injected with DEN (25 mg/kg). Nine months after DEN injection, mice were analyzed. Upper panel—representative liver images. Bottom panel—representative H&E stained tumor section (arrows indicate tumors). **d** Images of liver sections showing BrdU incorporation (left panel), and quantification (right panel). Scale bars: 100 µm (20× objective). Results are presented as the mean ± SEM percentage of positively stained cells from four section fields. ***p < 0.001 versus *HK2* ^F/F^/, by Student’s *t*-test. **e** Quantification of tumor incidence as measured by the number of tumors greater than or equal to 1.0 mm. The number of mice in each group is indicated. *p < 0.05 versus *HK2* ^*F/F*^, by Student’s *t*-test

A major enzymatic metabolic change that occurs in HCC is the isoform switch of the enzyme that catalyzes the first committed step in glucose metabolism from glucokinase to hexokinase 2^3^ (Supplementary Fig. 1). Analysis of HK2 expression in a tumor tissue microarray (TMA) of 312 samples derived from 153 human patients revealed high levels of HK2 expression in dysplasia and carcinoma and lower levels in cirrhosis, suggesting that the onset of HK2 expression occurs during or after cirrhosis^8^ (Fig. 1a, b). Detailed histopathological analyses showed significantly higher expression of HK2, as measured by IHC, in dysplasia and HCC than in cirrhosis and normal liver areas (Fig. 1a, *p* ≤ 0.05 by Student’s *t*-test). Within the HCC cells, the poorly differentiated cells expressed the highest level of HK2; within the poorly differentiated cells, the pleomorphic cells expressed the highest level of HK2 (Fig. 1b). This trend was observed in all patient samples tested regardless of the cause^8^. Therefore, if HK2 is required for HCC, targeting HK2 would selectively affect liver cancer cells without affecting normal hepatocytes. Indeed, genetic ablation of HK2 in liver-specific HK2 knockout (KO) mice (*HK2* ^*f/f*^*; AlbCre* mice) decreased proliferation and the incidence of tumors induced by diethylnitrosamine (DEN) (Fig. 1c, e).

### The effect of HK2 depletion on tumorigenesis and metabolism of HCC cells

**Fig. 2:**
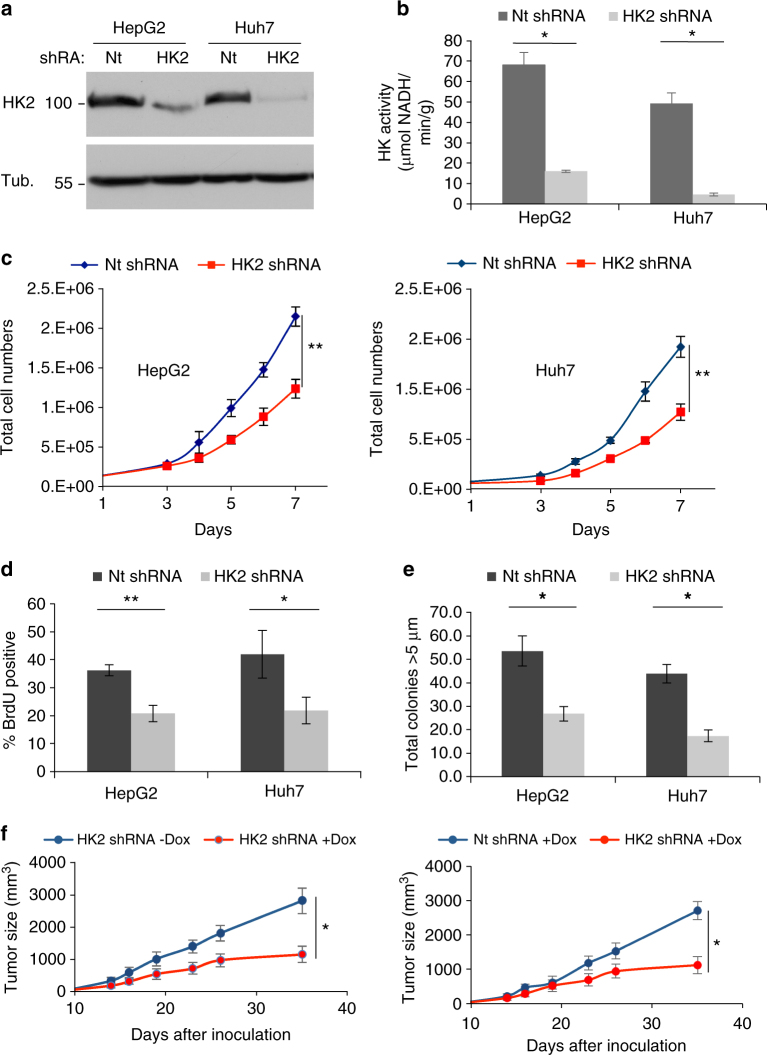
Effect of HK2 knockdown on tumorigenicity of human HCC cells. **a** Immunoblot analysis of inducible HK2 KD in HepG2 and Huh7 cells. **b** In vitro hexokinase activity assays upon inducible HK2 KD. **c** Cell proliferation curves. **d** BrdU incorporation analysis. Bar graphs show the percentage of BrdU-positive cells. **e** Anchorage-independent growth on soft agar. The cells were exposed to Dox for 3 days prior to analysis and maintained in Dox throughout the analyses. Bar graphs show the number of colonies larger than 5 μm. **f** In vivo tumor growth. Cells were inoculated into mice; after tumor detection, mice were fed either regular chow diet or with a Dox-infused diet. The left panel shows tumor growth of the Huh7 cells with inducible HK KD in either the absence or presence of Dox, and the right panel shows the tumor growth of the inducible HK KD in the presence of Dox compared to tumor growth of control cells in the presence of Dox (*n* = 6). Nt and HK2 shRNA indicate Dox-inducible non-targeting and HK2 shRNA, respectively. See Methods for experimental details. The results represent mean ± SEM, *p < 0.05, **p < 0.01, by Student’s *t*-test

HCC cell lines stably expressing doxycycline (Dox)-inducible HK2 shRNA or control shRNA were established as previously described^9^. Because of the reported Dox effect on metabolism and proliferation^10^, experiments included control cells expressing Dox-inducible non-target (Nt) shRNA in the presence of Dox. The reduction in HK2 protein levels following inducible knockdown (Fig. 2a) led to marked reductions in total hexokinase activity, confirming that the major hexokinase isoform expressed in these cell lines is HK2 (Fig. 2b). The displayed kinase activity in HepG2 cells was consistent with the higher level of HK2 protein observed in these cells compared to Huh7 cells. Loss of HK2 in HepG2 and Huh7 HCC cells led to reduced cell proliferation and tumorigenic potential. Proliferation was assessed by proliferation curve analysis and BrdU incorporation assays. Cells with inducible HK2 knockdown (KD) showed an approximately 50% reduction in proliferation in both assays (Fig. 2c, d). Cells with HK2 KD were also impaired in anchorage-independent growth (AIG); HepG2 cells exhibited a 50% decrease and Huh7 cells showed a 60% decrease (Fig. 2e). To test tumorigenic potential in vivo, Huh7 cells were used in subcutaneous tumorigenesis assays. Athymic-nude male mice were injected with either control (Nt) or HK2 Dox-inducible shRNA-expressing cells, and tumors were grown in the non-induced state (regular chow diet). After tumor formation, mice harboring Huh7 cells expressing control Nt Dox-inducible shRNA cells were provided a Dox-infused chow diet, while mice harboring Huh7 cells expressing HK2 Dox-inducible shRNA were provided with either a regular chow diet or a Dox-infused chow diet. Tumor size was tracked over approximately 3 weeks; mice containing the HK2 shRNA cells fed on the Dox-infused diet had significantly smaller tumors than control groups (Fig. 2f, *p* ≤ 0.05, by Student’s *t*-test). Reductions in tumor growth of about 50% were observed. These data support the role of HK2 as a tumor promoter and indicate a direct relationship between HK2 expression and cell proliferation.

**Fig. 3:**
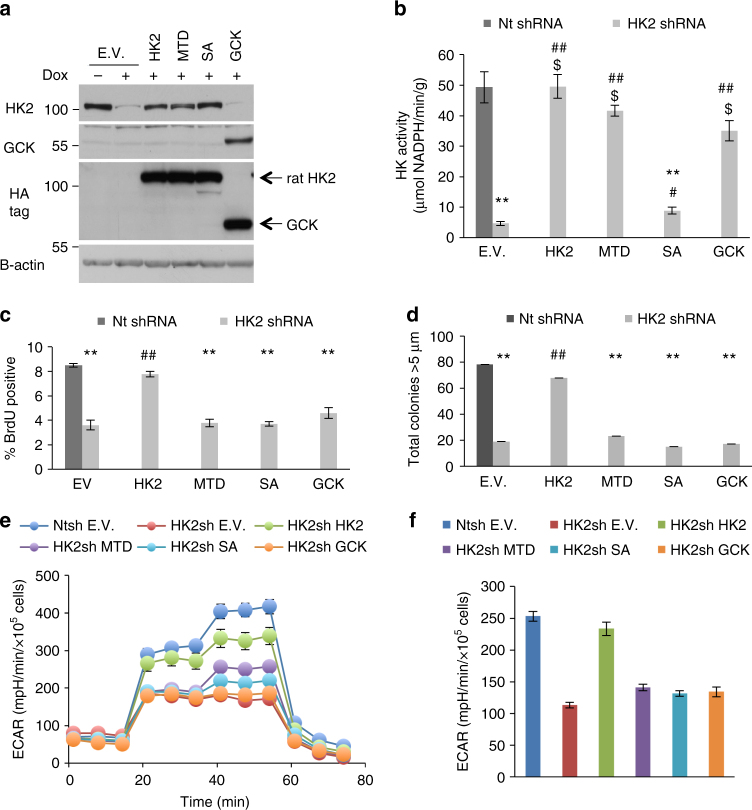
Replacement of endogenous HK2 with WT and HK2 mutants, and GCK. **a** Immunoblot analysis of engineered Huh7 cell lines for both endogenous HK2 KD and overexpression of different hexokinase isoforms. HA-tagged WT rat HK2 and mutants, resistant to silencing by human shRNA, and HA-tagged GCK were stably expressed in Huh7 cells expressing Dox-inducible HK2 shRNA. EV—empty vector, MTD—mitochondrial binding deficient mutant, SA—kinase dead mutant. **b** Analysis of in vitro hexokinase activity in the engineered stable cell lines. **c** Relative BrdU incorporation assay in the engineered cell lines. **d** AIG analysis in engineered cell lines. **e**, **f** Seahorse metabolic analysis (ECAR) of control cells (shNt EV), HK2 KD cells (shHK2 EV), shHK2 EV cells expressing WT HK2 (shHK2 HK2), expressing mitochondrial binding deficient mutant (shHK2 MTD), or glucokinase (shHK2 GCK). The cells were exposed to Dox for 3 days prior to analysis. The results are presented as the mean ± SEM; **p < 0.01 vs. Nt EV ^$^*p* > 0.07 vs. EV—Dox; ^#^*p* < 0.05, and ^##^*p* < 0.01 vs. EV + Dox, by Student’s t-test. Experiments were performed at least twice in triplicates

To determine the specificity of HK2 silencing and the role of HK2 activity in proliferation and oncogenesis, Rat WT HK2 and HK2 mutants, resistant to silencing, and human GCK were stably expressed in the cells carrying Dox-inducible shRNA (Fig. 3a, b and Supplementary Fig. 2a, b). The impaired proliferation and AIG after HK2 silencing could be restored by WT HK2 but not by a kinase dead mutant (SA) (Fig. 3c, d and Supplementary Fig. 2c). Interestingly, the mitochondrial binding deficient (MTD) mutant could not restore cell proliferation nor AIG. Ectopic expression of GCK also could not restore proliferation nor AIG in HK2-deficient cells (Fig. 3c, d and Supplementary Fig. 2c). These results indicate that first, both HK2 kinase activity and mitochondrial binding are required for cell proliferation and tumorigenesis, and second, GCK, with its high Km compared to HK2, cannot compensate for HK2 with its low Km. Thus, these results could explain why there is an isoform switch from glucokinase to HK2 during hepatocarcinogenesis.

To understand the mechanisms by which HK2 is required for proliferation, survival, and tumorigenesis of HCC cells, we analyzed the metabolic consequences of HK2 deficiency. Changes in glycolysis and respiration were measured after HK2 KD using the Seahorse metabolic analyzer. Measuring extracellular acidification rate (ECAR) with the Seahorse metabolic analyzer showed marked reductions in glycolytic function in both Huh7 and HepG2 cells upon HK2 loss compared to control cells (Fig. 3e, f and Supplementary Fig. 2d). Glycolysis (ECAR) can be restored by re-expressing WT HK2 but cannot be fully restored by either a mitochondrial binding deficient mutant (MTD) or GCK, which is consistent with the effect on AIG and proliferation. The results demonstrate that mitochondrial binding of HK2 is required for its full glycolytic function inside cells. Accordingly, we found that expressing WT HK2 or MTD-HK2 in M15-4 CHO cells, that have undetectable HK activity^11^, increased ECAR, but the increase induced by MTD-HK2 was about 40% less than the increase induced by WT HK2 despite similar HK activity in vitro (Supplementary Fig. 3).

**Fig. 4:**
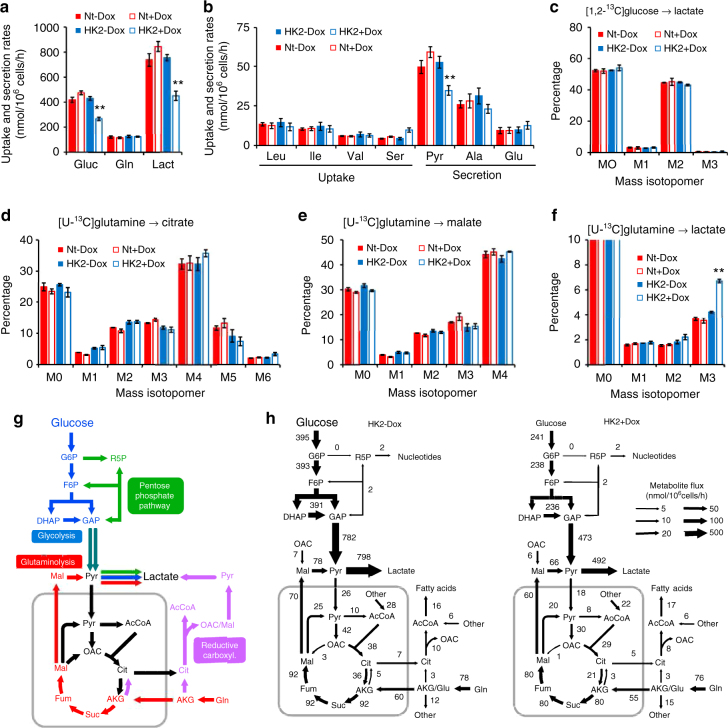
HK2 knockdown in Huh7 cells reduces glycolysis flux to lactate. **a** Glucose and glutamine uptake rates and lactate secretion rate in Huh7 cells expressing HK2-targeting shRNA (HK2), or non-targeting shRNA (Nt), under induced conditions (+Dox) or non-induced conditions (−Dox) (*n* = 8, biological replicates). **b** Uptake and secretion rates of pyruvate and amino acids (*n* = 8, biological replicates). **c** Mass isotopomer analysis of extracellular lactate in cultures with [1,2-^13^C]glucose and unlabeled glutamine (*n* = 2, biological replicates). **d** Mass isotopomer analysis of citrate in cells cultured with [U-^13^C]glutamine and unlabeled glucose (*n* = 2, biological replicates). **e** Mass isotopomer analysis of malate in cells cultured with [U-^13^C]glutamine and unlabeled glucose (*n* = 2, biological replicates). **f** Mass isotopomer analysis of extracellular lactate in cultures with [U-^13^C]glutamine and unlabeled glucose (*n* = 2, biological replicates). **g** Predominant metabolic pathways of glucose and glutamine metabolism in mammalian cells. **h** Metabolic flux distributions in Huh7 cells (HK2 - Dox) and Huh7 cells with HK2 knockdown (HK2 + Dox). Fluxes were determined by integrating uptake and secretion rates and isotopic labeling data from [1,2-^13^C]glucose and [U-^13^C]glutamine tracer experiments by metabolic flux analysis. Arrow widths indicate absolute magnitudes of net fluxes (nmol/10^6^ cells/h). Complete mass isotopomer distributions are shown in the Supplementary Data. All data represent the mean ± SD, **p < 0.001 by Welch’s unequal variances *t*-test

The effect of HK2 silencing on glycolysis was further analyzed in Huh7 cells by extracellular metabolite measurements and ^13^C-labeling techniques using [1,2-^13^C]glucose and [U-^13^C]glutamine as tracers. The glucose uptake rate and lactate secretion rate were both reduced by 40% after HK2 KD, while no significant effect on the glutamine uptake rate was observed (Fig. 4a, *p* ≥ 0.05, by Welch’s unequal variances t-test). We also did not find any increase in uptake rates of branched chain amino acids or secretion rates of alanine and glutamate (Fig. 4b and Supplementary Table 1); however, the pyruvate secretion rate was reduced by 35%. ^13^C-labeling analysis of extracellular lactate demonstrated that glucose was metabolized exclusively via glycolysis under all conditions (i.e., lactate was almost exclusively M2-labeled from [1,2-^13^C]glucose), with negligible flux through the pentose phosphate pathway (a low percentage of M1-labeled lactate; Fig. 4c). ^13^C-labeling analysis of intracellular metabolites demonstrated that the TCA cycle fluxes and relative influxes of glucose and glutamine into the TCA cycle were not affected by HK2 KD. As an example, there were no significant differences in the labeling of citrate and malate in experiments with [U-^13^C]glutamine (Fig. 4d, e, *p* ≥ 0.05, by Welch’s unequal variances t-test). The relatively low percentage of M5-labeled citrate from [U-^13^C] glutamine (~ 10%) demonstrated that glutamine was metabolized mainly via glutaminolysis and not via reductive carboxylation (Fig. 4d, g). ^13^C-labeling data also suggested that reductive carboxylation flux was unaffected by HK2 KD (i.e., no change in M5-labeled citrate from [U-^13^C]glutamine). ^13^C-labeling analysis of extracellular lactate from [U-^13^C]glutamine demonstrated that the relative contribution of glutaminolysis vs. glycolysis to lactate production was affected by HK2 KD; the percentage of M3-labeled lactate increased nearly 2-fold after HK2 KD (Fig. 4f). Given that glycolysis flux was reduced by nearly 2-fold after HK2 KD, the glutaminolysis rate was relatively constant (Fig. 4a).

**Fig. 5:**
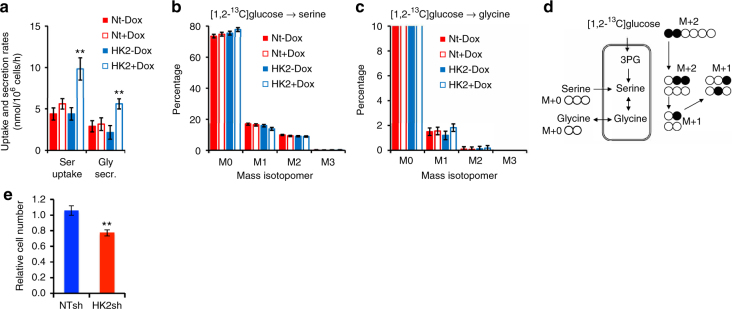
Changes in serine metabolism by HK2 loss. **a** Changes in extracellular serine uptake and glycine secretion after inducible HK2 KD (HK2 + DOX). **b** Tracing of [1,2-^13^C]glucose to serine. **c** Tracing of [1,2-^13^C]glucose to glycine. **d** Schematic showing [1,2-^13^C]glucose /serine/glycine tracing and serine/glycine exchange. All data represent the mean ± SD, **p < 0.001 by Welch’s unequal variances t-test. **e** Cell proliferation after inducible HK2 KD (HK2sh) or control (Ntsh) Huh7 cells in the presence or absence of serine. Cells were exposed to Dox for 4 days prior to analysis and maintained in Dox throughout the analyses. Bar graph shows relative cell number after 6 days of growth in serine-free media compared to cell number in complete media. The results represent mean ± SEM of two different experiments in triplicate, **p < 0.01 by Student’s *t*-test

We next performed metabolic flux analysis to better understand the metabolic consequences of HK2 KD. Steady state metabolic fluxes were determined by fitting the measured extracellular rates and mass isotopomer distributions of intracellular metabolites and external lactate to a compartmentalized metabolic network model (Supplementary Table 2 and Supplementary Data 1). For this analysis, the combined labeling data from four parallel labeling experiments with [1,2-^13^C]glucose and [U-^13^C]glutamine tracers (with two biological replicates for each tracer) were integrated (see Methods and Supplementary Data). We obtained statistically acceptable fits for all conditions. The flux analysis results quantitatively supported our conclusions from the ^13^C-labeling data analysis. Overall, the only significant impact of HK2 KD observed was reduced glycolysis flux, with no significant impact on other metabolic pathways, including the pentose phosphate pathway, glutaminolysis, reductive carboxylation, anaplerosis, cataplerosis and amino acid metabolism (Fig. 4h, *p* ≤ 0.05, by Welch’s unequal variances *t*-test). Notably, we also controlled for the potential Dox effect on metabolic fluxes (Supplementary Fig. 4 and Supplementary Data 1). Although we observed a modest increase in glucose flux by Dox in control cells, it did not change our overall conclusions. Interestingly, we observed a 2-fold increase in extracellular serine uptake with concomitant 2-fold increase in glycine excretion in HK2 KD cells (Fig. 5a), but we did not observe a change in the intracellular flux from glucose into the serine biosynthesis pathway (Fig. 5b, c). The results therefore suggest that as a consequence of HK2 silencing there is increased requirement for one-carbon contribution. The results also suggest that HK2 KD cells could be more vulnerable to extracellular serine deprivation. To test this possibility, we deprived the cells of extracellular serine, and found that the proliferation of HK KD cells was significantly decreased in comparison to control cells (Fig. 5e, *p* ≤ 0.05, by Student’s *t*-test).

**Fig. 6:**
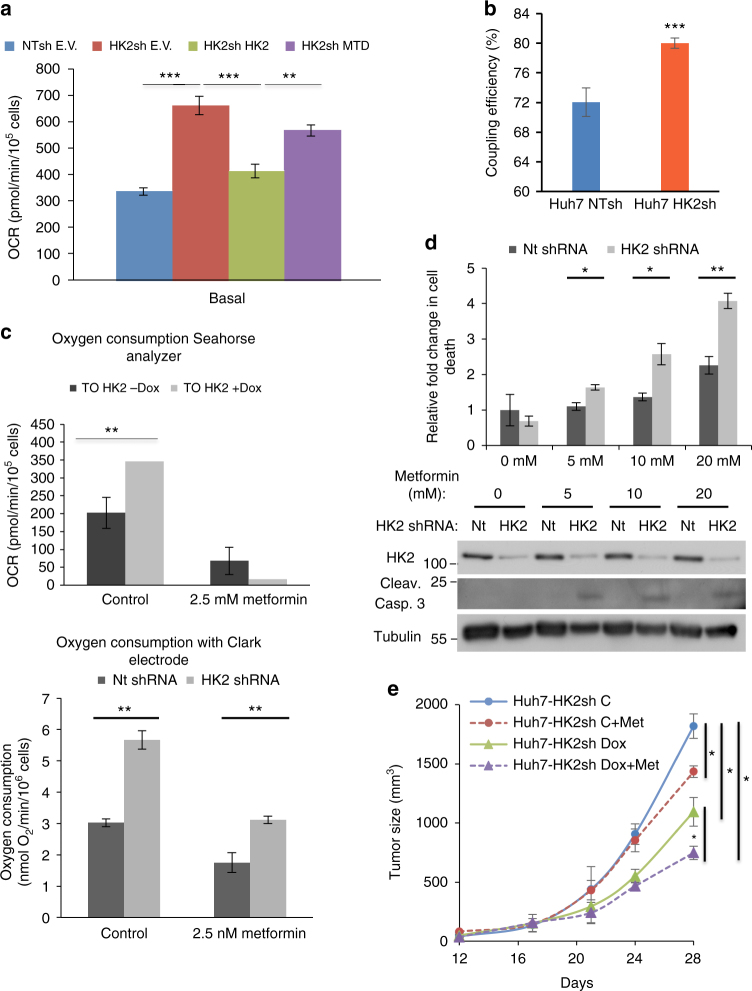
Effect of HK2 KD and metformin on Huh7 HCC cells. **a** Seahorse metabolic analysis of oxygen consumption rates (OCR). **b** Coupling efficiency as calculated from OCR data generated with Seahorse analyzer, as the ratio between ATP-linked respiration and basal respiration rate. Data are the average of two experiments done in quadruplet. **c** The effect of metformin on oxygen consumption as measured by the Seahorse analyzer (upper panel) and by the Clark electrode (lower panel). Cells were treated with the indicated concentrations of metformin for 24 h. **d** Cell death after HK2 KD in combination with metformin, upper panel—quantification of PI staining, lower panel—caspase 3 cleavage. **e** Huh7 cells with Dox-inducible HK2 shRNA were subjected to subcutaneous tumor growth. After tumor appearance, mice were exposed to either a Dox-infused diet alone, metformin alone or both. Tumor size was measured every 4 days, and tumor growth was plotted (*n* = 4 per each treatment). The results represent mean ± SEM, *p < 0.05, **p < 0.01 ***p < 0.005 by Student’s *t*-test

Analysis of the respiration rate following HK2 silencing showed an increased oxygen consumption rate (OCR) and respiration (Fig. 6a, c and Supplementary Fig. 5a, b), likely as a compensatory mechanism. Consistently, re-expression of WT HK2 completely restored OCR, whereas HK2-MTD mutant that did not fully restore ECAR (Fig. 3e, f) also did not fully restore OCR (Fig. 6a). Since we have not observed a change in the flux to the TCA cycle (Fig. 4) that could explain the increase in OCR, we calculated coupling efficiency. Coupling efficiency can be defined as the reciprocal relationship between oxidative phosphorylation and glycolysis adjusted to meet cellular energy needs and as calculated, coupling efficiency is significantly (*p* ≤ 0.05, by Student’s *t*-test) increased after HK2 KD. Energy production derived from glycolysis is decreased after HK2 KD and thus cells are adapting by increasing basal respiration and mitochondrial efficiency, hence at a steady state, a similar (slightly but not significantly higher) TCA flux (or NADH, FADH2 flux) can generate a better proton-motive force in the mitochondria. The energy generated by electron transport is then transferred more efficiently to ADP and can provide more energy to the cells and is sufficient to preserve energy homeostasis. Indeed we found that coupling efficiency is increased after HK2 KD in Huh7 cells (Fig. 6b), although it cannot fully explain the large increase in OXPHO.

In an attempt to further understand the increase in OXPHO despite no significant change in TCA flux, we further analyzed the ECAR and OCR data. The analysis of ECAR in Huh7 HK2 KD cells (Fig. 3e) shows that it is decreased by about 50% and that oligomycin stimulates ECAR in control cells but not in HK2 KD cells. Therefore, the basal glycolysis operates at full capacity in the HK2 KD cells. Analysis of OCR shows that basal respiration, as calculated by substracting non-mitochondrial from baseline respiration, is substantially increased in HK2 KD cells when compared to control cells. However both cell lines have similar maximal respiration rate upon addition of FCCP (Fig. 6a and Supplementary Fig. 5a, b). Thus, when spare capacity is calculated, we found that under basal conditions respiration in control cells operates at about 50% of their full capacity whereas HK2 KD cells do not have spare capacity suggesting that in HK2 KD cells respiration operates at full capacity (Supplementary Fig. 5c). These could explain, at least in part, the increased respiration in HK2 KD cells. Notably these explanations may apply to Huh7 cells but not necessarily to HepG2 cells (Supplementary Fig. 6a).

Because of the potential Dox effect on metabolism^10^, we included cells expressing Dox-inducible non-target shRNA as a control (Fig. 6a, d), but we found similar results in the absence of Dox in cells with the Dox-inducible HK2 shRNA (Fig. 6c). Since the loss of HK2 led to a reduction in glycolysis and a compensatory upregulation of oxidative phosphorylation, we hypothesized that targeting both metabolic pathways could potentially be therapeutic for HCC. Therefore, we employed metformin, a complex I inhibitor^12, 13^, to diminish the increase in respiration upon HK2 KD. Indeed, the increase in oxygen consumption was diminished after treatment with metformin, both in Huh7 and HepG2 cells (Fig. 6c and Supplementary Fig. 6b). Treatment with metformin in conjunction with HK2 silencing had a synergistic effect on cell death, as shown in Fig. 6d and Supplementary Fig. 6c. Accordingly, HK2 silencing and metformin treatment had a synergistic effect on tumor growth in vivo (Fig. 6e).

### The effect of HK2 depletion and metformin on mTORC1 activity

**Fig. 7:**
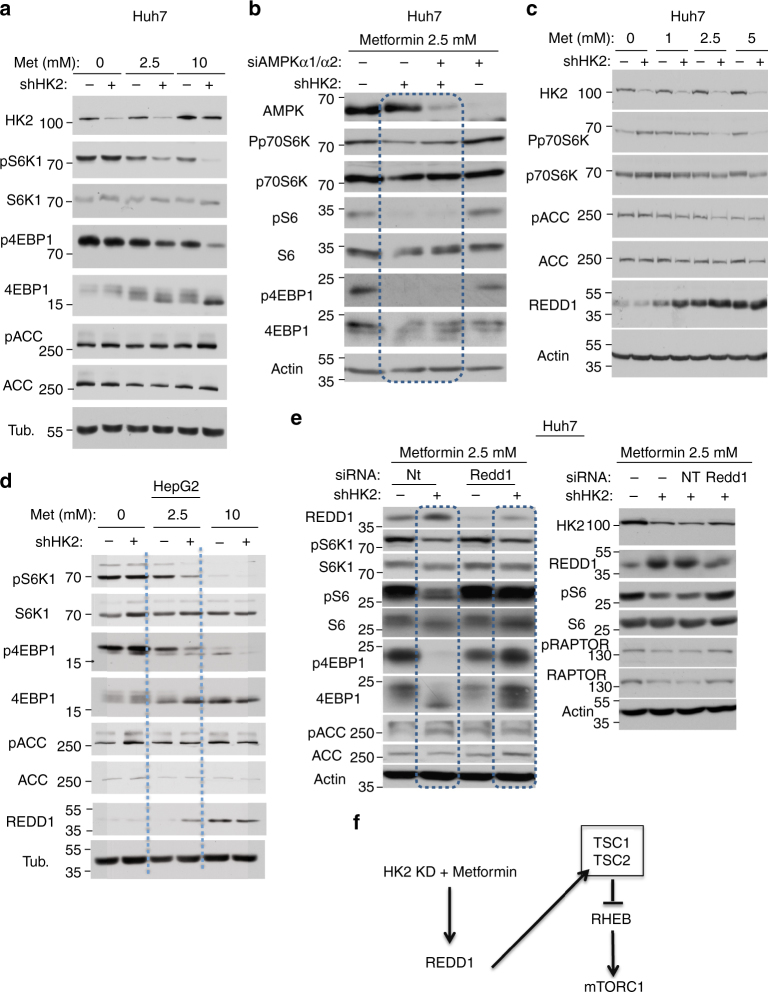
Effect of HK2 KD and metformin on mTORC1 activity in HCC cells. Cells were exposed to Dox for three days and metformin for the last 24 h. **a** Immunoblot showing the effect of HK2 KD and metformin on mTORC1 activity as measured by the phosphorylation of S6K1 and 4EBP1 and on AMPK activity as measured by the phosphorylation of ACC, in Huh7 cells. **b** Immunoblot showing the effect HK KD and metformin on mTORC1 activity after the KD of AMPKα1 and α2. **c** Immunoblot showing the induction of REDD1 expression after HK2 KD and metformin Huh7 cells. **d** Immunoblot showing the induction of REDD1 expression after HK2 KD and metformin HepG2 cells. **e** The effect of REDD1 KD on mTORC1 activity after HK2 KD and metformin as measured by S6K1 and 4EBP1 phosphorylation or S6 phosphorylation. AMPK kinase activity was evaluated by ACC phosphorylation (left panel) and by Raptor phosphorylation (right panel). Immunoblot images are representative of at least two independent experiments.** f** Schematic showing the mechanism by which the combination of HK2 KD and metformin inhibits mTORC1

Because depletion of HK2 together with metformin treatment is expected to increase energetic stress to activate AMPK, and subsequently to inhibit mTORC1 activity, we examined the effects of HK2 depletion and metformin treatment on AMPK and mTORC1 activity. Surprisingly, we found that neither HK2 depletion alone, nor a low concentration of metformin alone, nor HK2 depletion and metformin together significantly activated AMPK as measured by the phosphorylation of its substrate, ACC. However, we found that HK2 depletion and metformin have synergistic effects on mTORC1 activity as measured by S6K1 and 4EBP1 phosphorylation (Fig. 7a). The results suggest that the synergistic effect of HK2 depletion and metformin is AMPK-independent. Indeed, the depletion of AMPKα1 and AMPKα2 did not affect the inhibition of mTORC1 by HK2 depletion and metformin (Fig. 7b). However, we found a synergistic effect of HK2 depletion and metformin on the induction of REDD1 expression (Fig. 7c). REDD1 is known to interact with TSC2 and to increase the inhibitory effect of the TSC1/TSC2 heterodimer on mTORC1 activity^14, 15^. Similar results were obtained in HepG2 cells (Fig. 7d). To verify if REDD1 induction by HK2 depletion and metformin is required for the inhibition of mTORC1 activity, we depleted REDD1 using siRNA; the effect of HK2 depletion and metformin on mTORC1 activity was diminished (Fig. 7e). We concluded that the induction of REDD1 by HK2 depletion, together with metformin, contributes at least in part to the inhibition of mTORC1 activity (Fig. 7f).

### HK2 depletion sensitizes HCC cells to cell death and synergizes with sorafenib

**Fig. 8:**
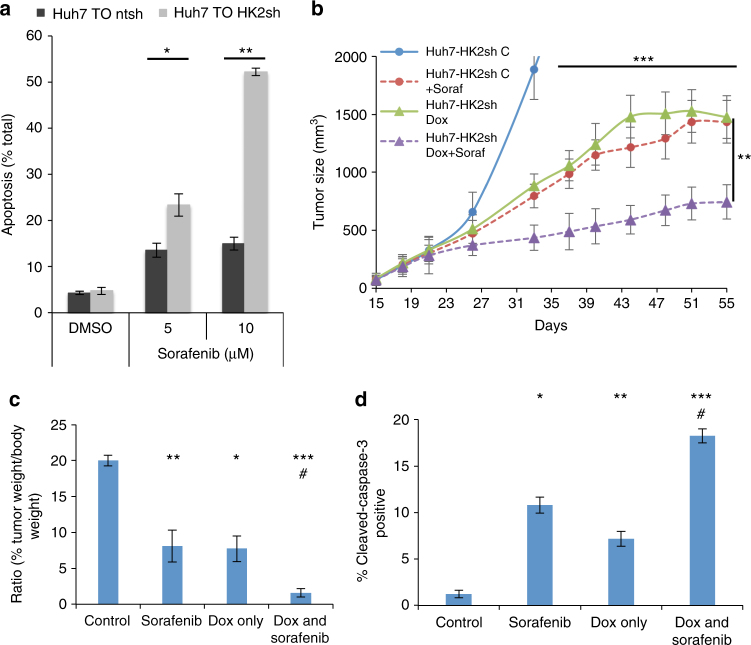
Effect of HK2 KD and sorafenib on cell death and tumor growth. **a** The effect of HK2 KD in combination with sorafenib on cell death of Huh7 cells. **b** Tumor growth in vivo of Huh7 cells expressing Dox-inducible HK2 shRNA in the absence or presence of a Dox-infused diet and in the presence or absence of sorafenib treatment. Note that monitoring the growth of tumors in control mice was stopped because once humane end-point criterion was reached the mice were killed. **c** Ratio of tumor to body weight at the end-point for control mice and at the last time point for treated mice. **d** Quantification of cleaved caspase 3 in the tumor sections. Results represent mean ± SEM, significance marked by * is to no Dox control and ^#^ to Dox control, */^#^*p* < 0.05, **p < 0.01, ***p < 0.001 by Student’s *t*-test

HK2 depletion sensitized HCC cells to cell death when subjected to low glucose or serum (Supplementary Fig. 7). Because HK2 silencing increased cell death induced by growth factor deprivation and because sorafenib, a multikinase inhibitor that inhibits both growth factor receptors and downstream kinases, is being used for HCC therapy, we examined the synergism between HK2 ablation and sorafenib. Indeed, we found that HK2 ablation sensitizes HCC cells to sorafenib (Fig. 8a). To determine whether sorafenib and HK2 ablation have a synergistic effect on tumor growth in vivo, Huh7 cells with Dox-inducible HK2 shRNA were inoculated into athymic-nude male mice. After tumor onset, mice were given either a regular chow diet or a DOX-infused chow diet with and without sorafenib injection. As shown in Fig. 8b and c, sorafenib alone and HK2 depletion alone significantly (*p* ≤ 0.05, by Student’s *t*-test) inhibited tumor growth. However, the combination of sorafenib and HK2 depletion decreased tumor growth much more substantially. Tumor sections were analyzed for cell death, using anti-cleaved caspase 3, in mice treated with sorafenib in combination with HK2 depletion and showed about 2-fold increases in cell death compared to mice with HK2 depletion alone or mice treated with sorafenib alone (Fig. 8d).

## Discussion

Most cancer cells reprogram cellular glucose metabolism to fulfill their anabolic demands. Among cancer cells, HCC cells probably display the most comprehensive reprogramming of glucose metabolism^3^. This is manifested by turning off the expression of certain enzymes required for the functionality of mature hepatocytes but not for HCC cells and by turning on the expression of enzymes that are required for the accelerated glucose metabolism in cancer cells. This metabolic distinction between HCC cells and normal hepatocytes has not yet been exploited to selectively target the cancer cells. A major distinction between HCC cells and normal hepatocytes is the enzymes that catalyze the first committed step in glucose metabolism. This step is catalyzed by GCK in normal hepatocytes, but GCK is suppressed in HCC cells; instead, HK2 expression is induced. HK2 expression is induced in many cancer cells. However, these cancer cells also express HK1, whereas human HCC cells generally do not express HK1. Because drugs that are delivered systemically tend to accumulate in the liver first, relatively low doses of HK2 inhibitors would selectively target HCC cells and not normal hepatocytes. Considering that systemic deletion of HK2 in mice does not elicit any severe physiological consequences, HK2 could be an excellent target for HCC therapy. We found that hepatic HK2 deletion inhibits hepatocarcinogenesis in mice and that its silencing in human HCC cells inhibits proliferation and tumorigenesis in vivo and increases sensitivity to cell death. Importantly, HK2 silencing synergized with metformin to inhibit tumor growth of human HCC cells. Since the metformin transporter is highly expressed in hepatocytes, the combination of HK2 inhibition and metformin might be an effective therapeutic avenue for HCC. Currently, the only FDA-approved therapeutic drug for HCC is sorafenib, but its efficacy in the treatment of HCC is relatively low. Our results that the combination of sorafenib and HK2 silencing markedly increased HCC cell death and synergistically inhibited tumor growth suggest that HK2 inhibition could significantly increase the efficacy of sorafenib. The studies described here showed that expression of GCK in HCC cells with HK2 KD did not restore ECAR and oncogenesis; this may explain why HCC cells induce the expression of HK2. Interestingly, a mitochondrial binding deficient mutant of HK2 did not restore ECAR and oncogenesis despite having similar in vitro kinase activity as WT hexokinase. Mitochondrial binding of hexokinases near VDAC may be required for access to ATP derived from OXPHO to efficiently phosphorylate glucose^16^. Our results provide experimental evidence for this hypothesis by showing that mitochondrial binding deficient HK2 cannot fully restore ECAR to the level of WT HK2. The MFA data showed that, despite markedly reduced glycolytic flux upon HK2 KD, glutamine flux did not significantly change. Interestingly, the flux to the oxidative and non-oxidative PPP did not change, although the mechanism is unclear. Overall, it appears that HCC cells are highly dependent on glutamine utilization for TCA flux. Notably, the importance of glutamine for tumor growth in vivo is controversial; glutamine utilization appears to be minimal in some in vivo tumors, except HCC tumors, which display high-glutamine utilization^17, 18^.

Although we did not find a significant change in the flux of glucose to the de novo serine biosynthesis pathway upon HK2 KD, the HK2 KD HCC cells increased the extracellular serine/glycine exchange by 2-fold. These results suggest that the HK2 KD cells require additional one-carbon units, which are generated during the conversion of serine to glycine. Consistently, we found that serine deprivation significantly reduced the proliferation of HK2 KD and not control cells. It is not clear why excess one-carbon units are required. It is tempting to speculate that it is required to generate NADH from the mitochondrial folate pathway to support the increase in OXPHO observed after HK2 KD. However, because the magnitude of the extra serine flux is small (~5 nmol/10^6^ cells/h) relative to OXPHO flux (~150 nmol/10^6^ cells/h), this might be unlikely.

Despite the lack of changes in the TCA cycle flux, we found an increase in OXPHO after HK2 KD, suggesting that OXPHO is increased independently of the TCA cycle. Although we don’t know the exact mechanism by which OXPHO is increased despite no change in the TCA flux, it could be partially attributed to an increase in coupling efficiency and the maximum use of respiration capacity in Huh7 HK2 KD cells. The increase in OXPHO was diminished if the cells were treated with metformin, which inhibits mitochondrial complex I. The combination of metformin and HK2 KD synergistically increased HCC cell death in vitro and tumor growth in vivo. The combination of metformin, which is approved for the treatment of diabetes, together with HK2 inhibition is an attractive approach for HCC therapy. Systemic administration of metformin primarily targets the liver;^13^ hepatocytes express relatively high levels of organic cation transporter (OCT1)^19^, the metformin transporter. HK2 is usually the only hexokinase expressed in HCC cells. Therefore, the combination of HK2 inhibition and metformin could selectively target HCC cells, especially since drugs that are systemically administered tend to accumulate in the liver first.

Our studies also uncovered a synergistic effect of HK2 KD and metformin on mTORC1 activity. Surprisingly, metformin alone in the range of 1–5 mM did not significantly affect AMPK and mTORC1 activities. However, the combination of HK2 KD and metformin inhibited mTORC1 activity, although it did not have a significant effect on AMPK activity. It is unclear why the combination of HK2 KD, which significantly decreased glycolysis, and metformin, which inhibits OXPHO, did not activate AMPK. One possibility is that the AMP/ATP and ADP/ATP ratios did not reach a threshold level sufficient to activate AMPK despite reduced glycolysis and OXPHO. Regardless, we observed that the combination of metformin and HK2 KD inhibited mTORC1 even when AMPK was depleted. Interestingly, it was previously reported that metformin inhibits mTORC1 by AMPK-independent mechanisms^20, 21^. We found that the combination of HK2 KD and metformin markedly increased the expression of REDD1, which inhibits mTORC1 by activating TSC2. Although we cannot completely exclude other mechanisms by which HK2 KD and metformin inhibit mTORC1, the attenuation of mTORC1 inhibition by REDD1 depletion suggest that this is a major mechanism. Notably, it was previously reported that REDD1 is induced by metformin in an AMPK-independent manner and in a p53-dependent manner^20^. However, we observed the induction of REDD1 even in Huh7 cells that carry a mutated p53^22^.

Finally, we found that HK2 KD in combination with sorafenib, the only FDA-approved drug for HCC, markedly increased HCC cell death and tumor growth in vivo. It remains to be determined whether small molecule inhibitors could be developed to inhibit specifically HK2. Both HK1 and HK2 are allosterically inhibited by their own catalytic product, G6P, but inorganic phosphate antagonizes the ability of G6P to inhibit HK1, and not HK2^5^. Thus, it might be possible to develop G6P mimetics to inhibit HK2 activity. Using this approach, it was recently reported that some glucosamine derivatives that bind both the G6P and glucose binding sites in hexokinase could preferentially inhibit HK2 activity^23^. Some of these compounds are two order of magnitude more potent inhibitors of HK2 than HK1. We used one of these compounds (compound 34) on Huh7 HCC cells and found that it markedly inhibited ECAR in these cells with no apparent inhibition in control Skhep1 cells (Supplementary Fig. 8). More studies are required to determine the stability of the compound in different cell type and the feasibility of its use in vivo in whole animal. Taken together, our studies showed that HK2 is an ideal therapeutic target for HCC either by itself or in combination with metformin or sorafenib.

## Methods

### Cell culture and stable cell line generation

All cell lines used were confirmed free from mycoplasma contamination, as determined by PCR. The human HCC cell lines HepG2 and Huh7 and lentiviral packaging line 293FT were grown in Dulbecco’s Modified Essential Media (DMEM, Hyclone), 10% fetal bovine serum (FBS, Atlanta Biologicals) or with TET-approved FBS (Atlanta Biologicals) (for inducible KD lines), and 1× Pen/Strep (Fisher) at 37 °C in a 5% CO_2_ atmosphere. Glucose concentrations were kept at either (25 mM) or (5.5 mM). Doxycycline induction was at (900 ng/mL) for both Huh7 and HepG2 inducible HK2 KD cell lines. For HK2 KD, stable expression of HK2-targeting shRNA was produced from either pLKO.1-Puro or Tet-On (T.O.)-pLKO.1-Puro (for inducible expression) lentiviral vectors (Invitrogen). The sequence targeting human HK2 used was: 5′-CCAAAGACATCTCAGACATTG-3′. The pLenti-6-D-TOPO-Blast lentiviral vector (Invitrogen) was used for the overexpression of hexokinases: Rat WT HK2, rat mitochondrial non-binding HK2 mutant (MTD: ∆1–20), rat catalytic HK2 mutant (SA: S155A/S603A), and human GCK (liver variant). Transduced cells were antibiotic selected with puromycin for 6–8 days or with blasticidin for 7–10 days. Cells were then removed from antibiotic selection and grown for generation of frozen stocks at earliest passage; all experiments were performed with early passage cells.

Transient transfection with REDD1 and AMPKα1/2 siRNA (Sigma) was employed to KD REDD1 and AMPK protein in Huh7 cells. Experiments were conducted by plating 8 × 10^5^ cells per 6-cm plate for next day transfection using Lipofectamine RNAiMax (Invitrogen). A final concentration of (50 µM) siRNA was used in culture media without antibiotics overnight. Cells were split the next day and treated with (2.5 mM) metformin the following day for 24 h before collecting.

### Proliferation assays

Proliferation rates were assessed in cell lines by proliferation curve analysis and BrdU incorporation assays. For both assays, cells were plated on 6-cm plates in triplicate (50 × 10^3^ for Huh7 and 75 × 10^3^ for HepG2). For proliferation curves, 3 days of growth was allowed before the first cell count (with the first count on the third day), and thereafter, counts were performed every other day for 3 total counts. Growth medium was replaced every two days to ensure no nutrient depletion would occur and/or to replace doxycycline for inducible lines. Total cell numbers of individual plates were determined by counting cells with a hemocytometer. Cell numbers for each time point were averaged and plotted by scatter plot with standard deviation or with standard error of the mean. In BrdU incorporation assays, growth was allowed for three days before labeling (labeled on third day) with (3 µg/mL) 5-Bromo-2′-deoxyuridine (BrdU) for 4 h. Cells were fixed with 95% ethanol under agitation. After a minimum of 24 h, cells were processed for immune detection of BrdU incorporation; DNA was denatured with (2 N) HCl, (0.5%) Triton X-100 for 1 h. Neutralization was done by adding (0.1 M) sodium tetraborate (NaB_4_O_7_ 10 H_2_O), pH 8.5, for 5 min. Cells were then washed, and an anti-BrdU antibody (1:500) was added for an overnight incubation at 4 °C. The next day, cells were washed and anti FITC-conjugated antibodies (1:250) were added and incubated for 2 h. Cells were then washed and resuspended with the fluorescent DNA stain, propidium iodide (PI), solution: PBS, (0.1%) Triton X-100, (0.2 mg/mL) RNaseA, and (0.05 mg/mL) PI and incubated for 30 min at room temperature. Samples were then diluted with 150 µL PBS, (10 mM) EDTA and filtered through FACS filter tubes (BD Bioscience). Samples were analyzed on a Beckman Coulter flow cytometer. For proliferation in serine-free media cells were exposed to Dox for 4 days prior to analysis and maintained in Dox throughout the analyses. On day 1, cells (90,000) were plated on 6-cm plates in triplicate. 24 h after plating, cells were washed twice with PBS and serine-free or complete media and dialyzed serum was added. On day 7, cells were washed once with PBS, trypsinized and counted by standard methods with a hemocytometer. Serine-free media (USBiological #D9802-01) was produced according to manufacturer’s protocol and supplemented with 5.5 mM glucose, 4 mM glutamine, 0.4 mM glycine, ±4 mM serine (for complete media).

### Anchorage-independent growth assay

Anchorage-independent growth assays were performed by plating 10–30 × 10^3^ cells in triplicate in 6-well plates in a mixture of media containing (0.35%) low-melt agarose over a base layer of (0.7%) low-melt agarose. Cells were grown for 3 weeks with media and Dox replacement every three days. At the end, the total colony number as well as all colonies larger than 5 µm were scored.

### Cell death assays

Cell death was assessed by either scoring apoptotic cells (cells with condensed or fragmented nuclei) visually at 20 × magnification after staining with the fluorescent DNA stain 4′,6-diamidino-2-phenylindole (DAPI) or by FACS using a flow cytometer after staining with PI. In these assays, cells were plated and subjected to individual treatment regimens. At the time of collection, cells were fixed with 4% formalin for over 24 h at room temperature and subsequently stained with DAPI and scored. For FACS analysis, culture media and washes were collected before trypsinization. Cells were fixed with 3 mL of (95%) ethanol under agitation. After a minimum of 24 h, cells were then washed and resuspended with the fluorescent DNA stain propidium iodide (PI) solution: PBS, (0.1%) Triton X-100, (0.2 mg/mL) RNaseA, and (0.05 mg/mL) PI and incubated for 30 min at room temperature. Samples were then diluted with 150 µL PBS, (10 mM) EDTA and filtered through FACS filter tubes (BD Bioscience). Samples were analyzed on a Beckman Coulter flow cytometer.

### Immunoblotting

To prepare samples, frozen cell pellets were lysed with lysis buffer: (20 mM) HEPES, (150 mM) NaCl, (1%) Triton X-100, (1 mM) EDTA, (1 mM) EGTA, (10 mM) sodium pyrophosphate, (100 mM) NaF, (5 mM) iodoacetic acid, (20 nM) okadaic acid, (0.2 mM) phenylmethylsulfonyl flouride (PMSF), complete protease inhibitor cocktail and phosphatase inhibitor cocktail tablets (Roche Diagnostics). Next, 20–40 µg protein suspended in SDS loading buffer was run on 6–12% SDS polyacrylamide gels and electrotransferred to nitrocellulose membranes. Immunoblotting was done per standard methods, using TBST and TBST, (5% BSA) as the wash and blocking/primary antibody dilution solutions, respectively. Antibodies were used at a 1:1 K dilution, unless otherwise stated, and included: anti-HK2 (Cell Signaling Technology, 2867), anti-HK1 (Cell Signaling Technology, 2024), anti-GCK, 1:500 (Santa Cruz, 7908), anti-HA (Convance, MMS101R), anti-B-actin, 1:2 K (Sigma, A5441), anti-phospho-ACC (Cell Signaling, 3661), anti-ACC, 1:500 (Cell Signaling, 3662), anti-phospho-Raptor (Cell Signaling 2083), anti-Raptor (Cell Signaling, 2280), anti-phospho-p70S6K (Cell Signaling, 9205), anti-p70S6K (Cell Signaling, 9202), anti-phospho-S6 Ribosomal Protein, 1:5 K (Cell Signaling, 2211), anti-S6 Ribosomal Protein, 1:50 K (Cell Signaling, 2217), anti-phospho-4EBP1 (S65) (Cell Signaling, 9451), anti-phospho-4EBP1 (T37/46) (Cell Signaling, 2855), anti-4EBP1 (Cell Signaling, 9452), anti-REDD1, 1:750 (Protein Tech, 10638–7AD), and anti-Cleaved Caspase 3, 1:500 (Cell Signaling, 9664). Membranes were incubated with 1:3000 dilutions of appropriate secondary antibodies for 1 h at room temperature, and the Pierce ECL Western Blotting Substrate (Thermo Scientific) was used for development of immunoreactive bands. Expanded image files are available in Supplementary Fig. 9.

For immunoblotting of tissue samples, proteins were extracted from paired liver samples collected from human patients and embedded in OCT, snap frozen in liquid nitrogen, and stored at −80 °C. As the research involved analysis of de-identified tissue samples no patients consent was required as established by the Office for the Protection of Research Subjects. After excess OCT was scraped from the sample using a sterile scalpel blade, liver sample were scraped into a tube containing ice-cold PBS and rinsed twice. Then protein lysis buffer (described above) was added to samples. Samples were vortexed, homogenized and subjected to three freeze/thaw cycles in dry ice, centrifuged and supernatant collected, and stored at −80 °C for subsequent immunoblotting.

### Hexokinase activity assay

Hexokinase activity was measured indirectly as a readout of NADPH production, a byproduct of the reaction converting G6P to 6-phosphoglucolactone by the enzyme glucose-6-phosphate dehydrogenase (the first step of the pentose phosphate pathway), by spectrophotometry. Whole-cell lysates were collected in a mixture of (45 mM) Tris-Cl, pH 8.2, (50 mM) KH2PO_4_, (10 mM) glucose, (11.1 mM) monothioglycerol, (0.5 mM) EDTA, and (0.2%) Triton X-100 and incubated on ice for 1 h with intermittent vortexing to shear cells. To conduct the in vitro hexokinase assay, 20 µL of whole-cell extract was added to an assay mixture of (41.7 mM) Tris-Cl, pH 8.5, (7.7 mM) MgCl_2_, (4.2 mM) glucose, (10.6 mM) monothioglycerol, (0.5 mM) EDTA, (0.05%) Triton X-100, (1 mM) NaPO_4_, (45 mM KCl), (0.5 mg/mL) NADP, and (6.7 mM) ATP and mixed. Absorbance was then measured at 340 nm wavelength and was considered the baseline measurement. Then, (1 U/mL) glucose-6-phosphate dehydrogenase was added and absorbance measured every 2.5 min, for three measurements. Measurements were used to generate a logarithmic curve, and the hexokinase activity was determined. Values were normalized to the protein concentration of the lysates.

### Metabolomic assays

For Seahorse assays, 1.5 × 10^4^ cells per well were plated onto Seahorse 96-well plates and both Glyco and Mito Stress Tests were performed following the manufacturer’s specifications. Media contained either (5 or 25 mM) glucose or (4 mM) glutamine without sodium pyruvate. Assays were normalized to total cell numbers, counted the next day. For labeling experiments, 7.0 × 10^5^ cells were plated onto 6-cm plates for next day pulse-labeling with either 5 mM [1,2-^13^C]glucose or 4 mM [U-^13^C]glutamine (Cambridge Isotope Laboratories). Parallel labeling experiments were performed for 20 and 24 h to validate that isotopic steady state was reached. Extra plates were included for cell counts for all time points, including the starting point. For each of the labeled plates, 200 µL of sample media was collected and stored at −80 °C for all time points, including the starting point. At the time of collection, plates were washed twice with 5 mL of (9 g/L) NaCl. Then, cells were incubated with 1.5 mL of ice-cold methanol for 5 min, scraped down with cell scrapers, and transferred into glass centrifuge tubes (Kimble Chase). Next, 1.5 mL of chloroform was added to the tubes and vortexed on high speed for 10 s. Finally, 1.5 mL of sterile water was added and the tubes were vortexed on high speed for 1 min, capped, and stored at 4 °C overnight. The next day, samples were centrifuged at 2000 RPM for 20 min at 4 °C. Then, 3 mL of the aqueous phase was transferred to two 1.5-mL centrifuge tubes, dried completely under nitrogen gas at 37 °C, and stored at −80 °C. Finally, 1.5 mL of organic phase was transferred to new glass centrifuge tubes and stored at −80 °C. Samples were subjected to GC/MS analysis.

### Metabolite analysis

Concentrations of glucose and lactate were measured by a YSI 2700 biochemistry analyzer (YSI, Yellow Springs, OH). For quantification of other extracellular metabolites, isotope ratio analysis was applied^24^, where 100 µL of culture medium was supplemented with internal standards: 15 µL of 10 mg/mL of [U-^13^C]algal hydrolysate (Cambridge Isotope Laboratories); 15 µL of 50 mM [U-^13^C]glutamine (or 50 mM unlabeled glutamine for analysis of ^13^C-glutamine labeling experiments); 15 µL of 10 mM [U-^13^C]pyruvate; and 15 µL of 0.1 N NaOH. Samples were then deproteinized with 300 µL of cold acetone (−20 °C), centrifuged, and the liquid was evaporated to dryness under nitrogen gas flow at 37 °C. The dried samples were derivatized as described below in preparation for GC/MS.

### Isotopic labeling analysis

Isotopic labeling of extracted intracellular metabolites and external lactate was determined by GC/MS (PLoS One, 10:^12^ e0145850, 2015). The dried intracellular metabolites were first dissolved in 50 µL of 2 wt% methoxylamine hydrochloride in pyridine and incubated at 37 °C for 90 min on a heating block. Next, 80 µL of N-methyl-N-(tert-butyldimethylsilyl)-trifluoroacetamide (MTBSTFA) + 1% tert-butyldimetheylchlorosilane (TBDMCS) (Thermo Scientific, Bellefonte, PA) was added and the samples were incubated for 30 min at 60 °C. After an overnight incubation at room temperature, the derivatized samples were briefly centrifuged and the clear liquid was transferred into GC vials for GC/MS analysis. GC/MS analysis was performed on an Agilent 7890B GC system equipped with a DB-5MS capillary column (30 m, 0.25 mm i.d., 0.25-µm phase thickness; Agilent J&W Scientific), connected to an Agilent 5977 A Mass Spectrometer operating under ionization by electron impact (EI) at 70 eV. Mass isotopomer distributions were obtained by integration^25^ and corrected for natural isotope abundances^26^.

### Metabolic flux analysis (MFA)

Steady state metabolic fluxes were determined by fitting extracellular rates (glucose/glutamine uptake, lactate/pyruvate/alanine/glutamate secretion) and mass isotopomer distributions of intracellular metabolites and external lactate to a compartmentalized metabolic network model using the Metran software^27^. The metabolic network model describes the stoichiometry and carbon atom transitions for reactions in central carbon metabolism and amino acid metabolism (Supplementary Materials). The model contains three distinct metabolic compartments: extracellular, cytosol, and mitochondrion. The metabolites pyruvate, acetyl-CoA, citrate, α-ketoglutarate, malate, oxaloacetate, alanine, glutamate, and aspartate are metabolically active in both the cytosol and mitochondrion. During the extraction process, intracellular pools of metabolites are homogenized. As such, the measured isotopic labeling of metabolites reflects the mixture of distinct metabolic pools. The MFA model contains mixing reactions to account for mixing of mitochondrial and cytosolic metabolite pools during extraction^28^. For MFA, labeling data from four parallel labeling experiments, i.e., with [1,2-^13^C]glucose (20-h and 24-h time points) and [U-^13^C]glutamine (20-h and 24-h time points), were fitted simultaneously to the same network model to estimate intracellular fluxes^29^. All data used for flux calculations are reported in the Supplementary Materials. To ensure that the global best solution was identified, flux estimation was repeated at least 50 times starting with random initial values. At convergence, a chi-square test was applied to test the goodness-of-fit, and accurate 95% confidence intervals were calculated by determining the sensitivity of the sum of squared residuals to flux parameter variations^30^. The complete flux results, including upper and lower bounds of 95% confidence intervals for all fluxes, are reported in Supp. Table 3.

### In vivo subcutaneous xenograft tumorigenesis assay

Athymic male nude mice were purchased from Charles River Laboratories and were between 6 and 8 weeks old at the time of experiments. Cells were grown in culture before collection for injection subcutaneously into the left and right flanks of each mouse (0.5–1.0 × 10^6^ in 100 µL per site). Once tumors were ~65 mm^3^ volume, mice from each group were randomly split into two groups for treatment with a (200 mg/kg) doxycycline diet (Bioserve) or a regular chow diet. Tumor size was tracked manually for five weeks, with measurements every 7–14 days, where total volume was determined to be the sum of *a* × *b* × *c*, with *a* = longest length of diameter, *b* = shortest length in diameter, and *c* = the length of the height. End points were reached, and mice were killed once the tumor size measured 2 cm. Quantification of cleaved caspase 3-positive cells in tumor sections was done as previously described^31^. All animal experiments were approved by the University of Illinois at Chicago Institutional Animal Care and Use Committee, as required by United States Animal Welfare Act, and the NIH’s policy.

### Diethylnitrosamine (DEN)-induced hepatocarcinogenesis

DEN-induced hepatocarcinogenesis was performed by intraperitoneal injection of (25 mg/kg body weight) into 14-day-old male mice^31^. Livers from nine-month-old DEN treated mice were analyzed macroscopically for tumor lesions. For tissue section analyses and BrdU incorporation, nine-month old DEN treated mice were injected intraperitoneally with BrdU (Sigma) in PBS (50 mg/kg of body weight), 2 h prior to killing and tissue collection. Livers were then collected fixed in 10% formalin for 18 h and subsequently preserved with 70% ethanol. Fixed tissues were then processed and embedded in paraffin. Paraffin embedded tissues were processed and 5 μM slides were prepared for hematoxilin and eosin (H&E) and BrdU staining. After dewaxing and rehydration, proteinase K was used for antigen retrieval. Slides were then incubated with anti-BrdU mouse monoclonal (Dako# M0744) and detection was done by M.O.M.-DAB kits (Vector Laboratories). For quantification, cells were counted from four high staining section fields at a 20 × magnification using eight mice per group.

### Statistical analysis

Unless otherwise indicated, statistical analysis was done with unpaired Student’s *t*-tests, or Welch’s unequal variances *t*-test for the flux analyses with SD or SEM, as indicated in the figure legend. Sample sizes were chosen to satisfy statistical power based on previous experience and knowledge. To this end, each experiment was run in triplicate in at least two independent sets. For animal studies, we performed power analyses using a web-based tool at www.biomath.info. Within group, variation is expected to be about 30% at the end-point and we are expecting a difference of at least 50% between two groups. On the basis of those eight tumors/group will have sufficient power with a confidence level of 0.95. The only data excluded from studies were in mice where tumors failed to grow or any technical replicates that were extreme outliers from experiments with large sample sizes (*n* = 6). The only experiments that required randomization were the in vivo studies, where by groups were assigned to provide the most consistency in tumor size across groups at the start of the experiment. Blinding in in vivo experiments was not done during experimentation, but during analysis of data., i.e., IHC slides, by covering the label during analysis. Statistics for each experiment were justified as appropriate, because the data does meet the assumptions of the test. Estimates of variation are based on individual tests and were within limits, and the variance between groups compared were similar.

### Data availability

All data generated or analyzed during this study are included in this published article and its Supplementary Information files or from the corresponding author upon reasonable request.

## Electronic supplementary material

Supplementary Information

Description of Additional Supplementary Files

Supplementary Data 1
