# Author Correction: Hexokinase-2 depletion inhibits glycolysis and induces oxidative phosphorylation in hepatocellular carcinoma and sensitizes to metformin

**DOI:** 10.1038/s41467-018-04182-z

**Published:** 2018-06-26

**Authors:** Dannielle DeWaal, Veronique Nogueira, Alexander R. Terry, Krushna C. Patra, Sang-Min Jeon, Grace Guzman, Jennifer Au, Christopher P. Long, Maciek R. Antoniewicz, Nissim Hay

**Affiliations:** 1 Department of Biochemistry and Molecular Genetics, College of Medicine, University of Illinois at Chicago, Chicago, IL 60607 USA; 2 Research & Development Section, Jesse Brown VA Medical Center, Chicago, IL 60612 USA; 3 Department of Pathology, College of Medicine, Cancer Center, University of Illinois Hospital and Health Science Chicago, Chicago, IL 60612 USA; 4 Department of Chemical and Biomolecular Engineering, Metabolic Engineering and Systems Biology Laboratory, University of Delaware, Newark, DE 19716 USA; 5 Present Address: Massachusetts General Hospital Cancer Center, Harvard Medical School, Boston, MA 02114 USA; 6 Present Address: College of Pharmacy, Ajou University, Yeongtong-gu, Suwon-siGyeonggi-do, 443-749 Korea

Correction to: *Nature Communications* 10.1038/s41467-017-02733-4, published online 31 January 2018.

In the originally published version of this Article, the colours of the bars in Fig. 4b were inadvertently switched during the production process, such that ‘HK2-Dox’ and ‘HK2+Dox’ were depicted in red and ‘Nt-Dox’ and ‘Nt+Dox’ were depicted in blue. These errors have now been corrected in both the PDF and HTML versions of the Article.

