# Supplementary material for: Hexokinase-2 depletion inhibits glycolysis and induces oxidative phosphorylation in hepatocellular carcinoma and sensitizes to metformin
Source: Nat Commun. 2018 Jan 31;9:446. doi: 10.1038/s41467-017-02733-4 (PMC5792493; doi:10.1038/s41467-017-02733-4)
Supplement: Supplementary file 1 — Supplementary Information [file 41467_2017_2733_MOESM1_ESM.pdf]

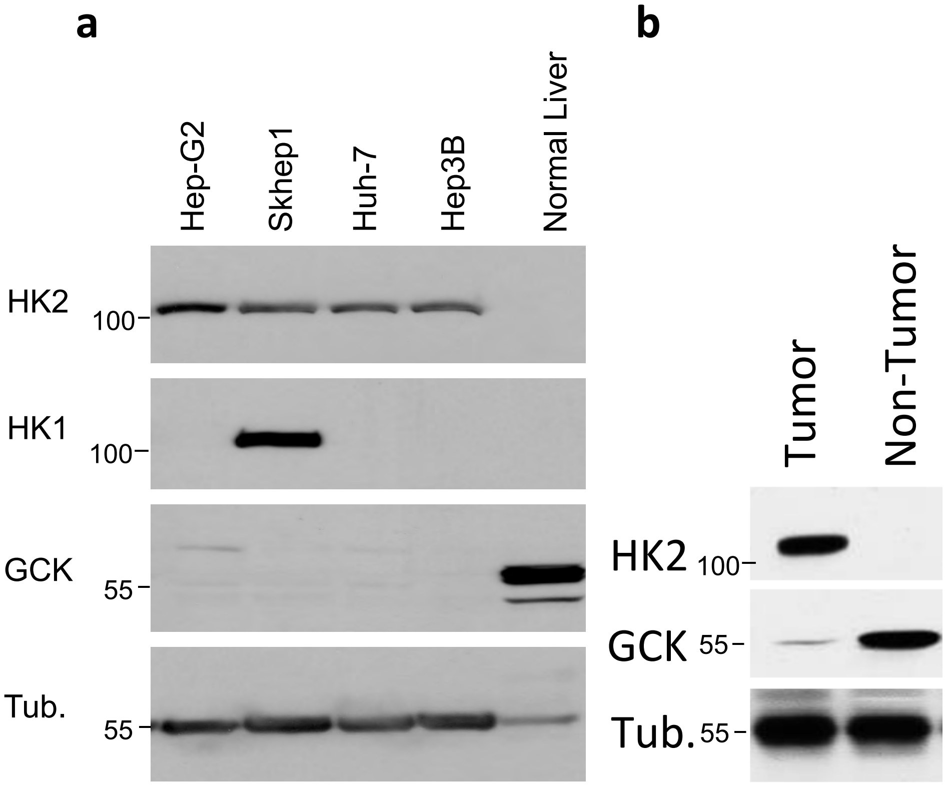

Supplementary Figure 1: Hexokinase isoform switch in HCC. a In human HCC cell lines: HepG2, Huh7, and Hep3B, HK2 is the only isoform expressed, with no expression of HK1 or GCK. The endothelial tumor cell line, Skhep1, and mouse normal liver tissue were used as positive controls for expression of HK1 and GCK r espectively. **b** Isoform switch during mouse hepatocarcinogenesis whereby GCK, the normal adult hepatic hexokinase isoform, is downregulated, and HK2 is upregulated.

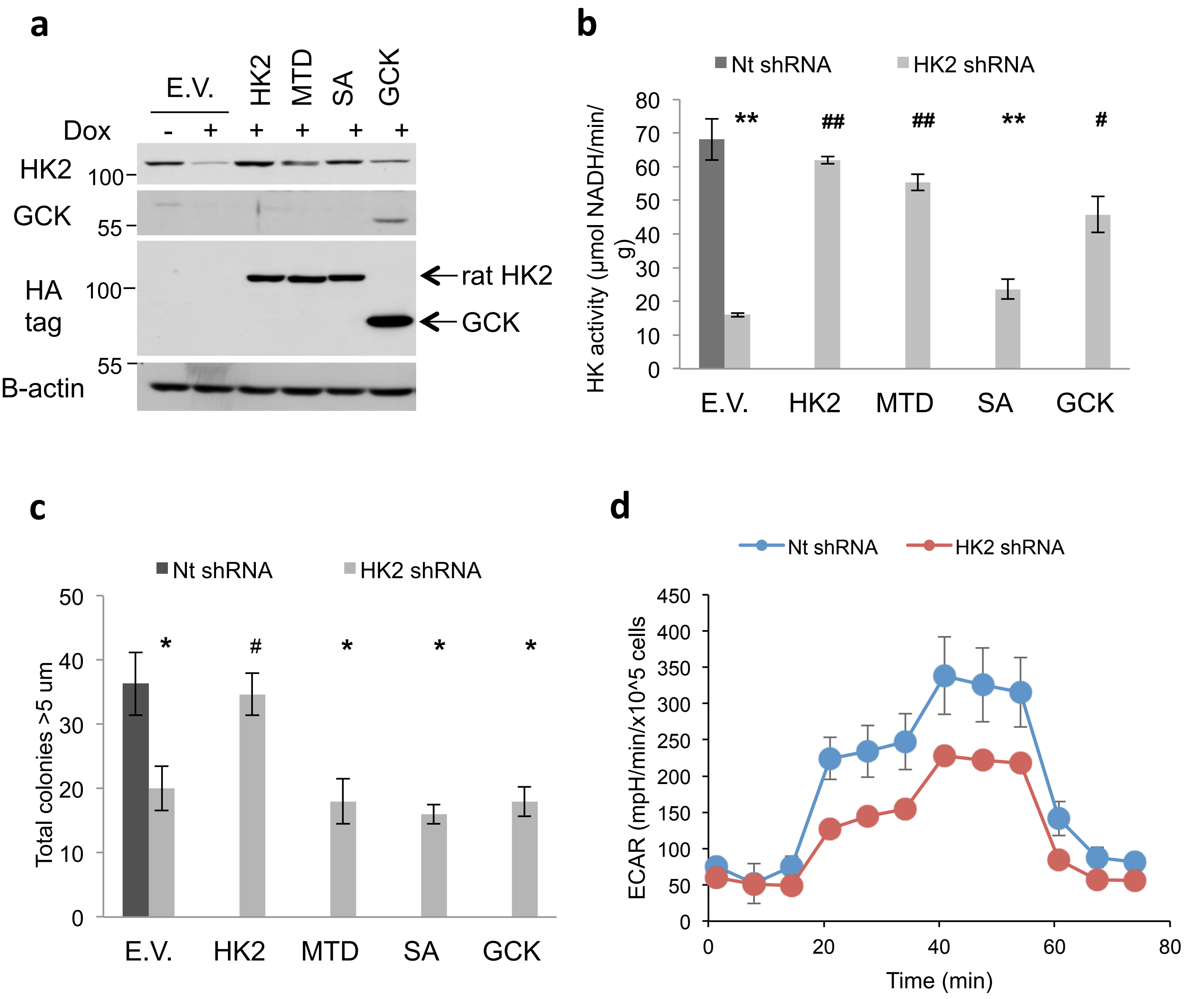

Supplementary Figure 2. Replacement of endogenous HK2 with WT and HK2 mutants, and GCK in HepG2 cells.

a Immunoblot analysis of engineered HepG2 cell lines for both, endogenous HK2 KD, and overexpression of different hexokinase isoforms. HA-tagged WT rat HK2 and mutants, resistant to silencing by human shRNA, and HA-tagged GCK were stably expressed in HepG2 cells expressing Dox-inducible HK2 shRNA. E.V. – empty vector, MTD- mitochondrial binding deficient mutant, SA-kinase dead mutant. **b** Analysis of in vitro hexokinase activity in the engineered stable cell lines. **c** AIG analysis in engineered cell lines. The cells were exposed to Dox for 3 days prior to the analysis. **d** Glycolysis (ECAR) after the knockdown of HK2Results represent mean +/- SEM of at least two independent experiments in triplicat es.**p<0.01 vs. Nt E.V; #p<0.05, and ##p<0.01 vs. E.V. +Dox.

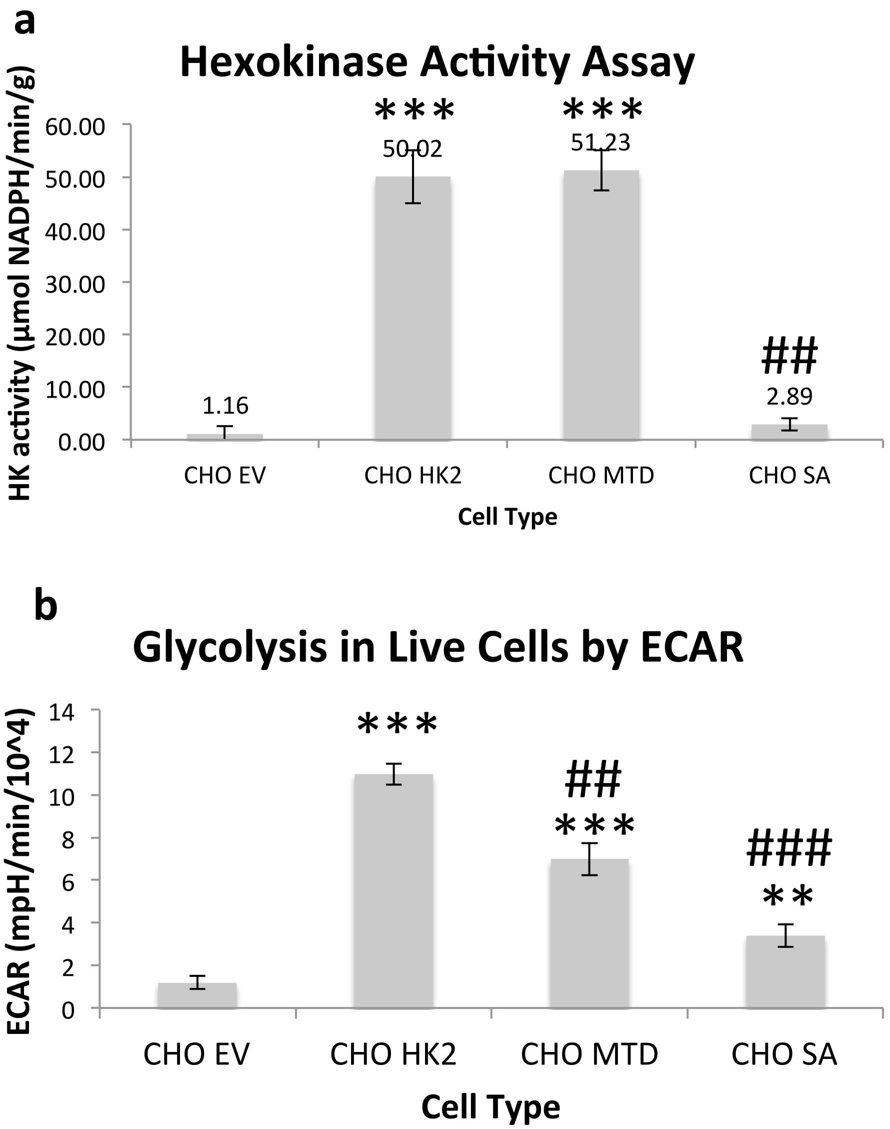

Supplementary Figure 3: The mitochondrial binding domain of HK2 is required for efficient glycolysis.
Empty vector (EV) lentivirus or lentiviruses expressing WT, mitochondrial binding deficient mutant (MTD), and catalytically inactive mutant (DA) of HK2 were used to infect M15-4 CHO cells, lacking hexokinase activity, and generate stably expressing cell lines. **a** Hexokinase activity in cell extracts isolated from the different cell lines. **b** Glycolysis in the different cell lines as measured by the Seahorse metabolic analyzer. The results represent mean +/-SEM of three independent experiments. * $P < 0.05$, ** $p < 0.005$, ***$p < 0.0005$ vs. EV, And # $P < 0.05$, ## $P < 0.005$, ### $P < 0.0005$ vs. WT HK2.

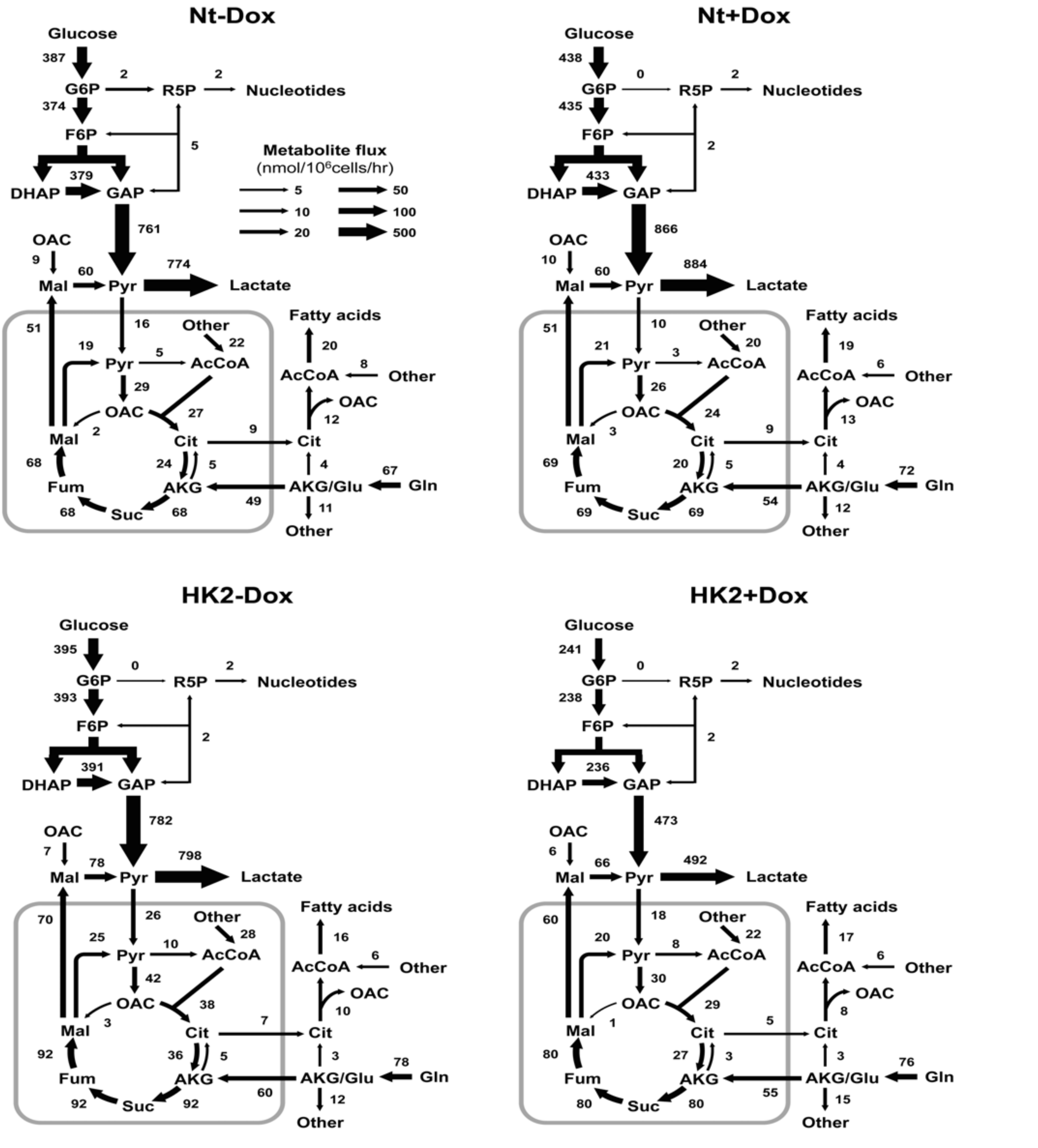

Supplementary Figure 4: Metabolic flux analyses in the presence or absence of Dox in Huh7 cells expressing non-target (Nt) or HK2 shRNA. Fluxes were determined by integrating uptake and secretion rates and isotopic labeling data from [1,2-^{13}C]glucose and [U-^{13}C]glutamine tracer experiments by metabolic flux analysis. Arrow widths indicate absolute magnitudes of net fluxes (nmol/10^6 cells/hr).

All data represent mean ± s.d. **P < 0.001, Welch's unequal variances t-test.

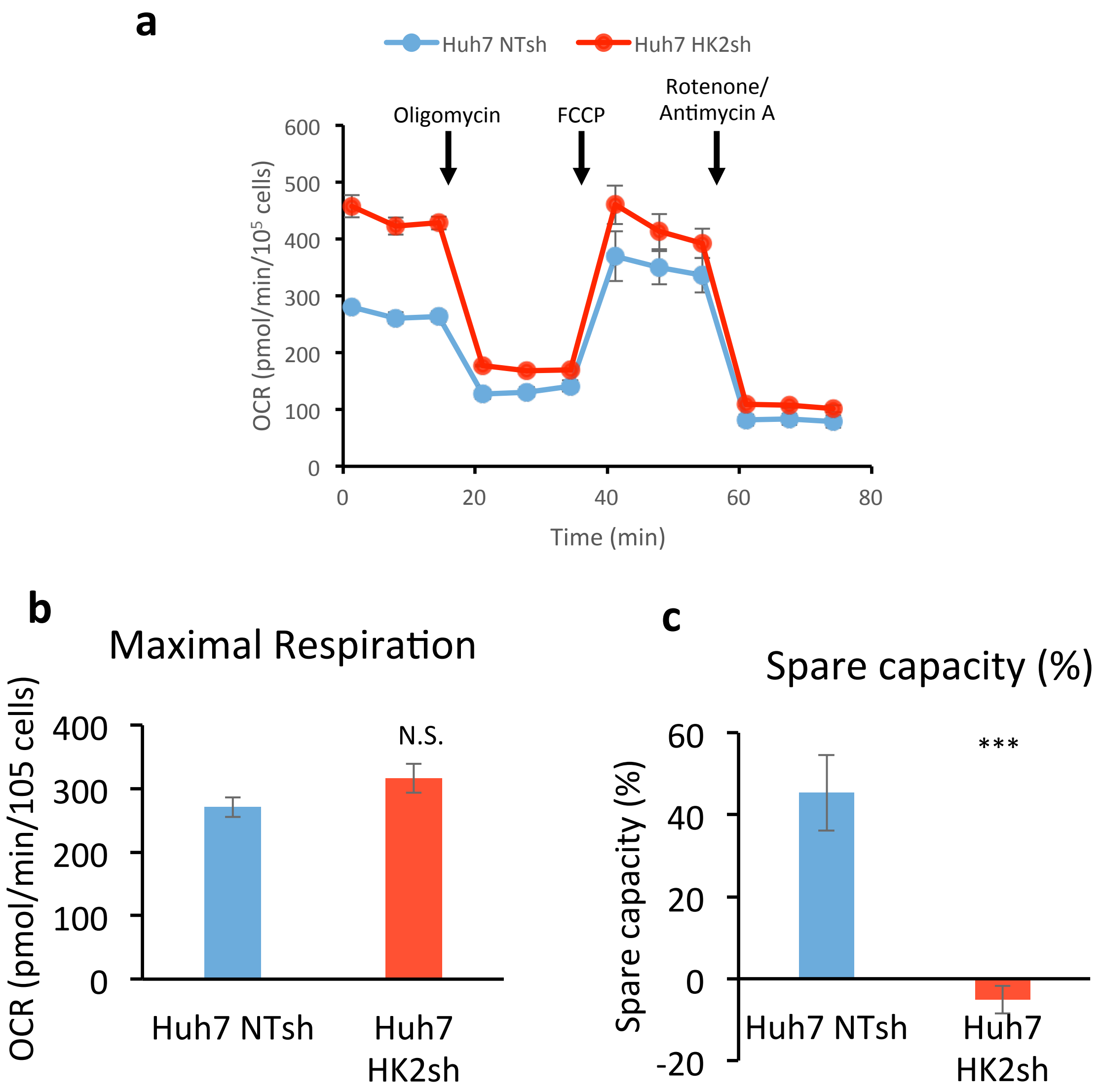

Supplementary Figure 5: OCR and respiration capacity in Huh7NTsh and Huh7 HK2 KD cells.

a Seahorse metabolic analysis of oxygen consumption rates (OCR) in Huh7 NTsh and Huh7 HK2sh cells. Traces are the average of two experiments done in Quadruplet and data collected used for calculation of basal respiration (Fig. 6a), coupling efficiency (Fig. 6b), maximal respiration and percentage of Spare capacity.

b Maximal respiration calculated as the difference between maximum OCR obtained after the addition of FCCP (collection points 7-9) and non-mitochondrial OCR FCCP (collection points 10-12).

c Spare capacity calculated as the ratio between maximal and basal respiration. All data are the average of two experiments done in quadruplet.

N.S. Non-Significant; *** $p<0.001$

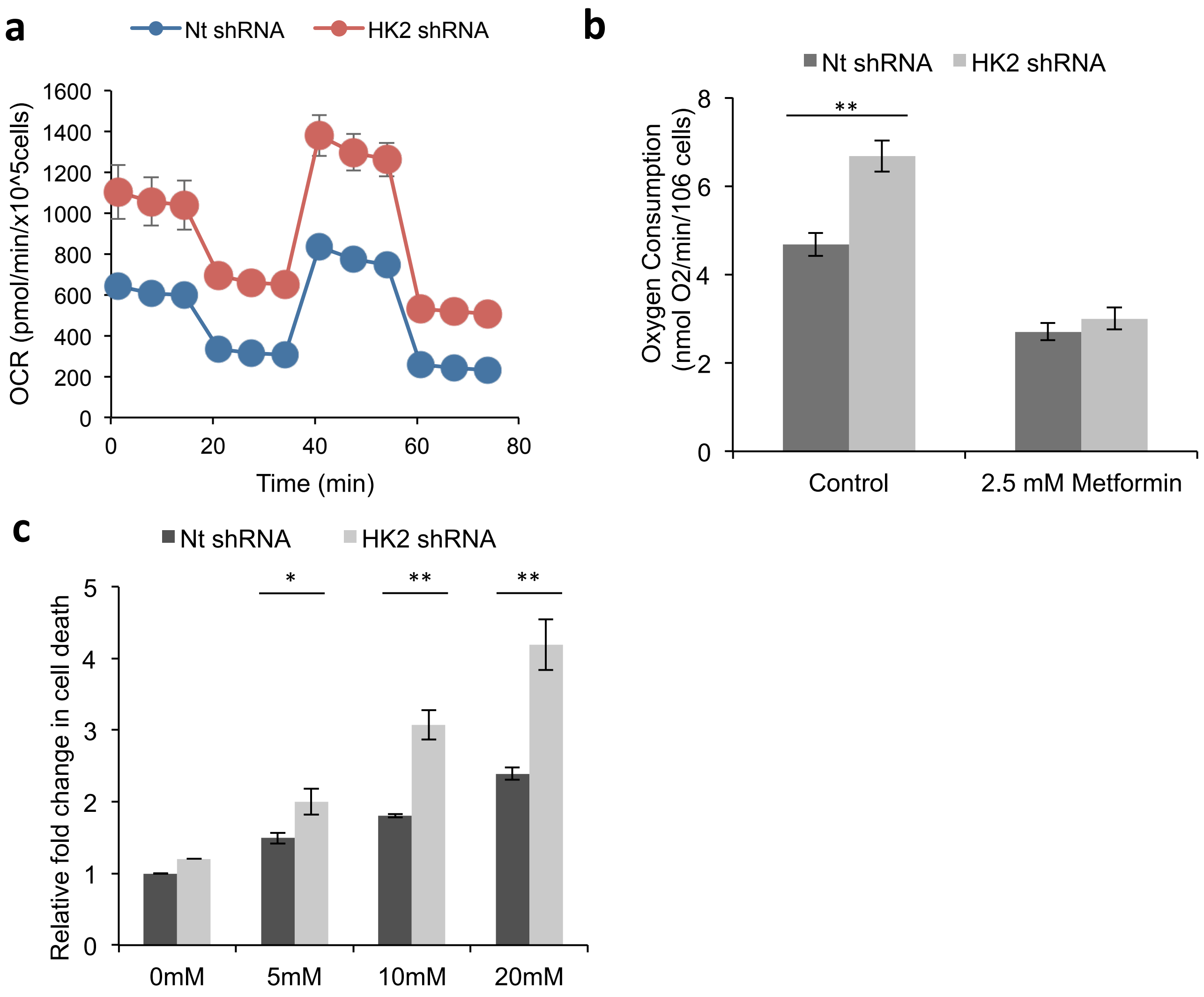

Supplementary Figure 6. The effect of HK2 KD and metformin on re spiration and cell death of HepG2 HCC cells. a Seahorse metaboli c analysis of oxygen consumption rates (OCR). **b** The effect of metformi n on oxygen consumption as measured by the Clark electrode (lower pan el). Cells were treated with the Indicated concentrations of metformin for 24 h. **c** Cell death after HK2 KD in combination with metformin. The results repres ent mean +/-SEM. *p<0.05, **p<0.01.

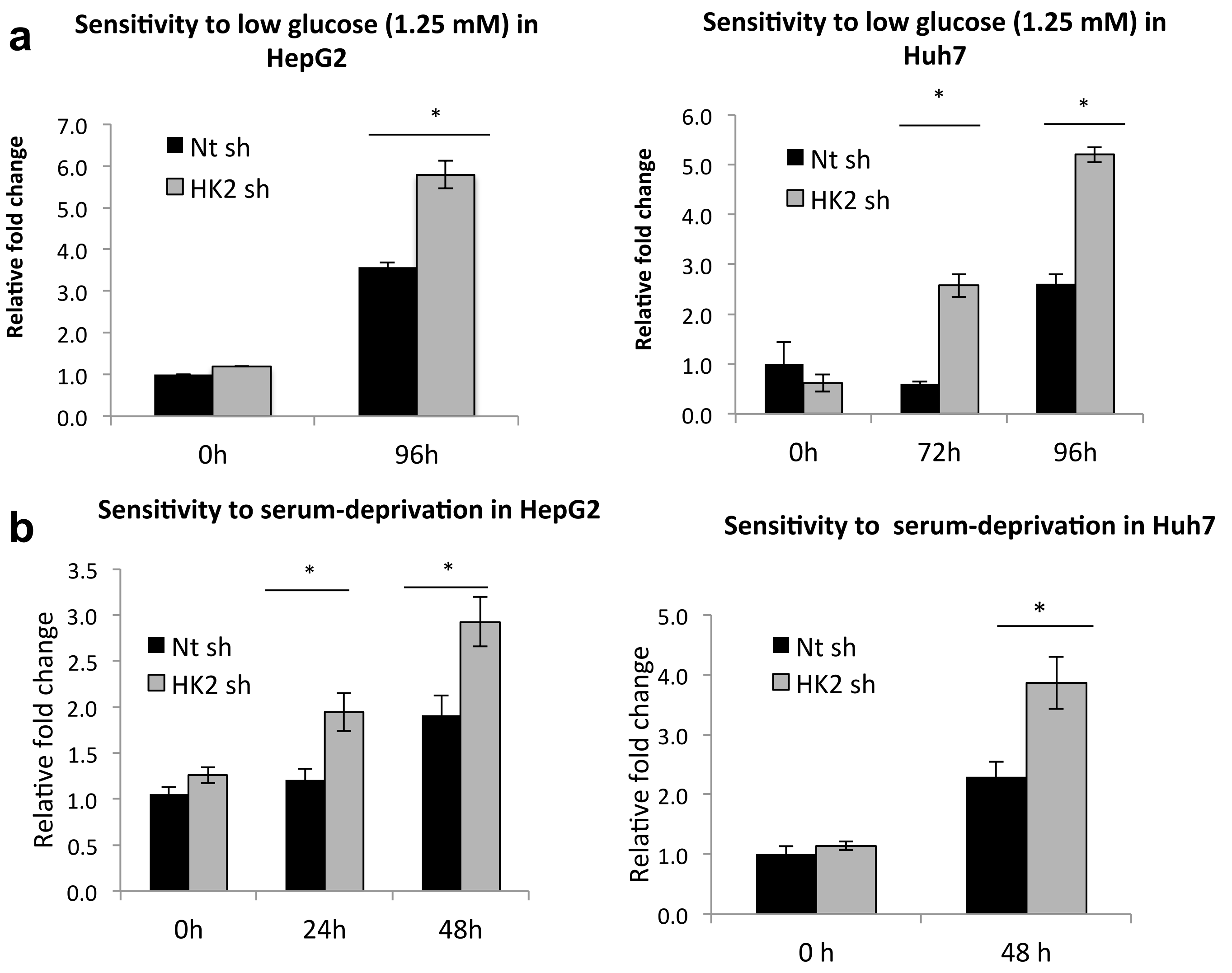

Supplementary Figure 7: Loss of HK2 sensitizes HCC cells to cell death.
Cells were treated with Dox and exposed to low glucose or serum deprivation. At the indicated time points cell death was measured and quantified.
(**a** and **b**) Loss of HK2 in HepG2 and Huh7 HCC cells increased susceptibility to cell death by low glucose (a) and serum deprivation (b). The results represent mean +/- SEM of three independent experiments in triplicates. *P≤0.01

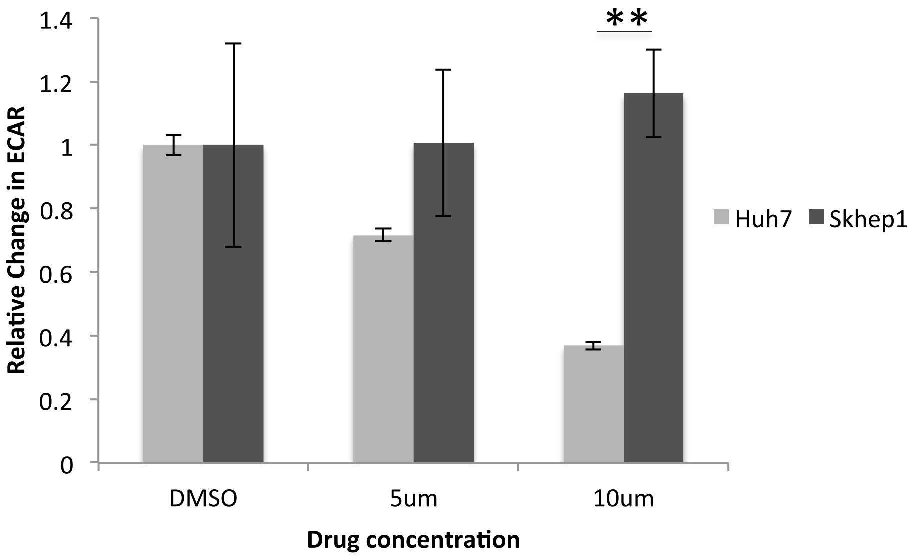

Supplementary Figure 8. An HK2-specific inhibitor decreased ECAR in Huh7 cells but not Skhep1 cells. Cells were pre-incubated for 2 hours with compound 34, before undergoing a standard glucose stress test by seahorse analyzer. Due to low amount of drug provided, this experiment is is 2 wells for each drug treatment. The results represent mean +/-SEM. **p<0.005.

Supplementary Figure 9. Uncropped Immunoblot images

Figure 2a

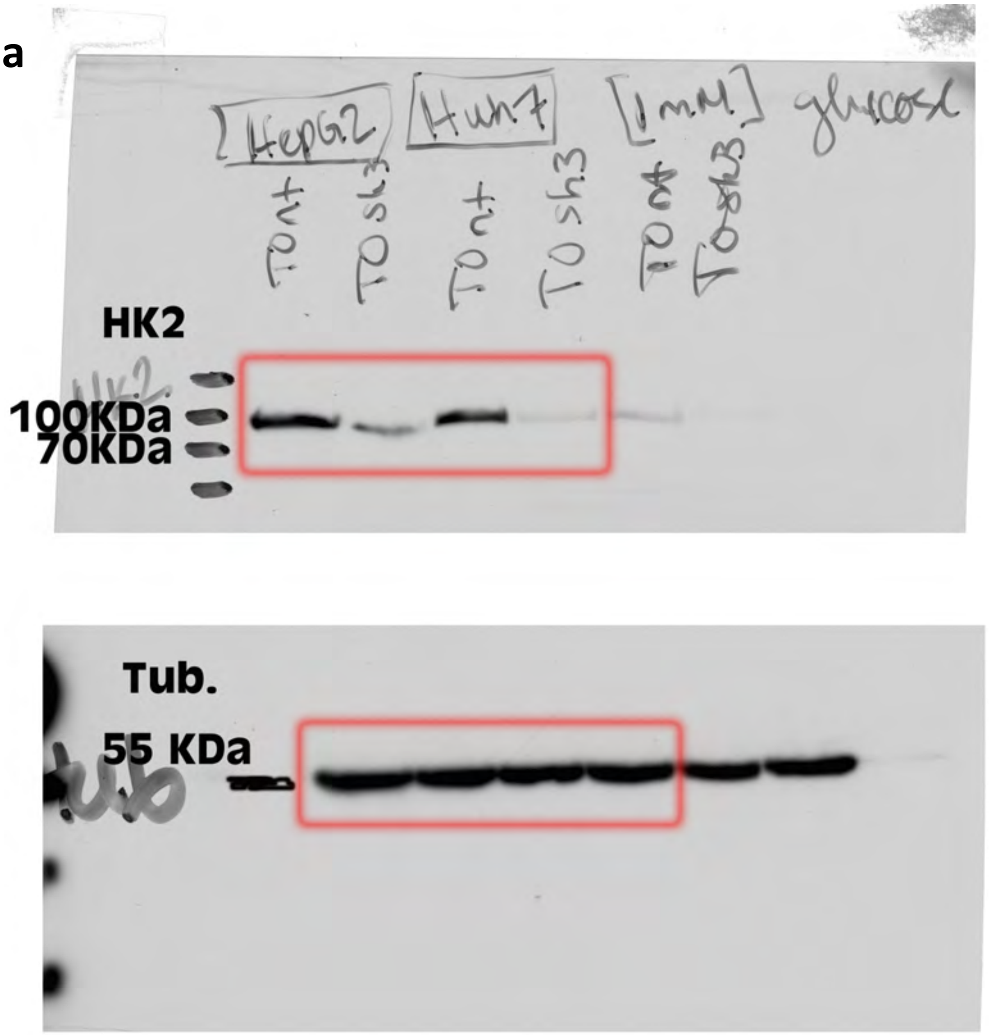

Supplementary Figure 9. Uncropped Immunoblot images

Figure 3a

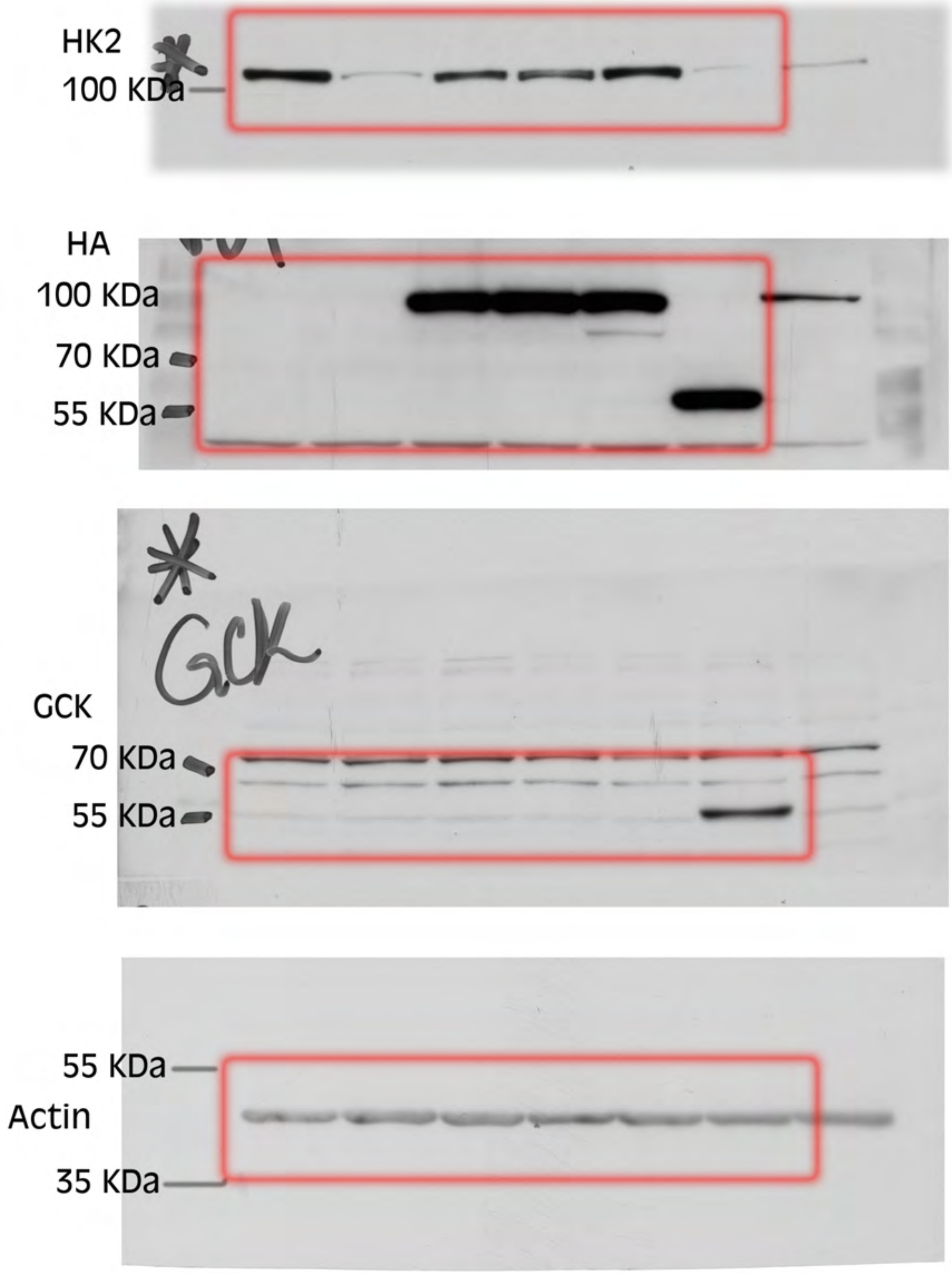

Supplementary Figure 9. Uncropped Immunoblot images

Figure 5c

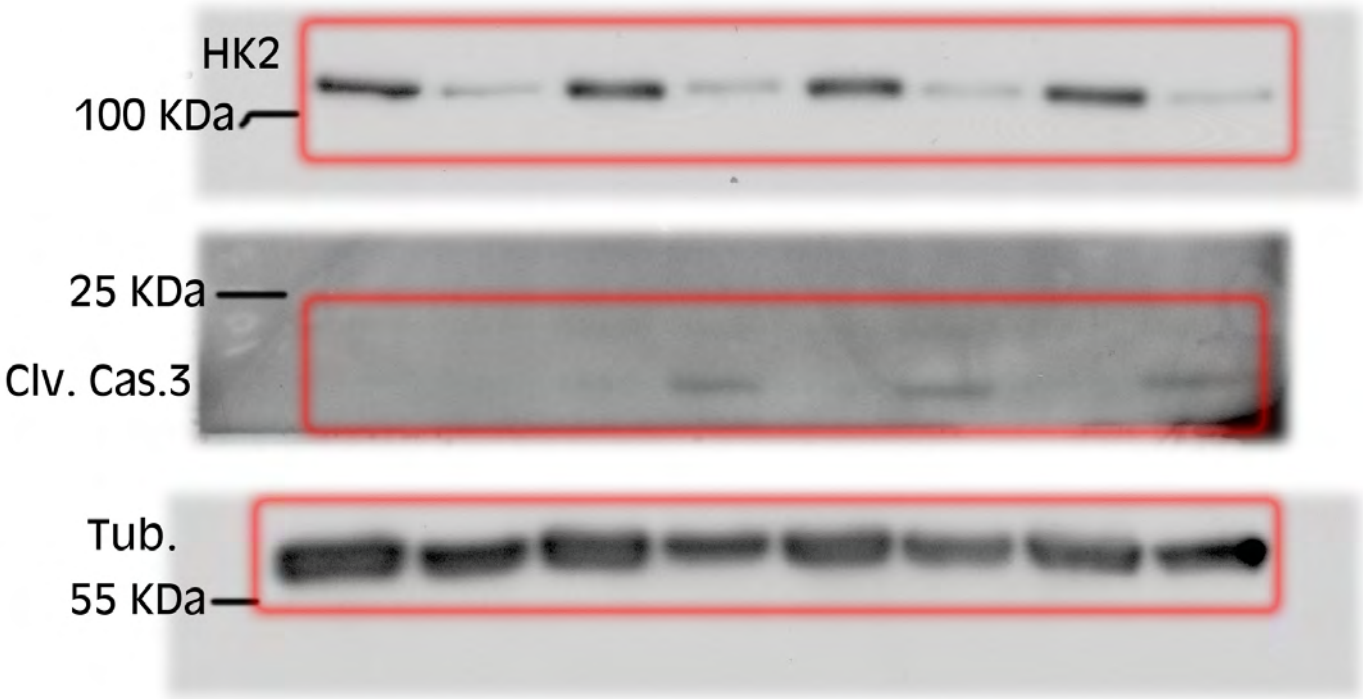

Supplementary Figure 9. Uncropped Immunoblot images

Figure 6a

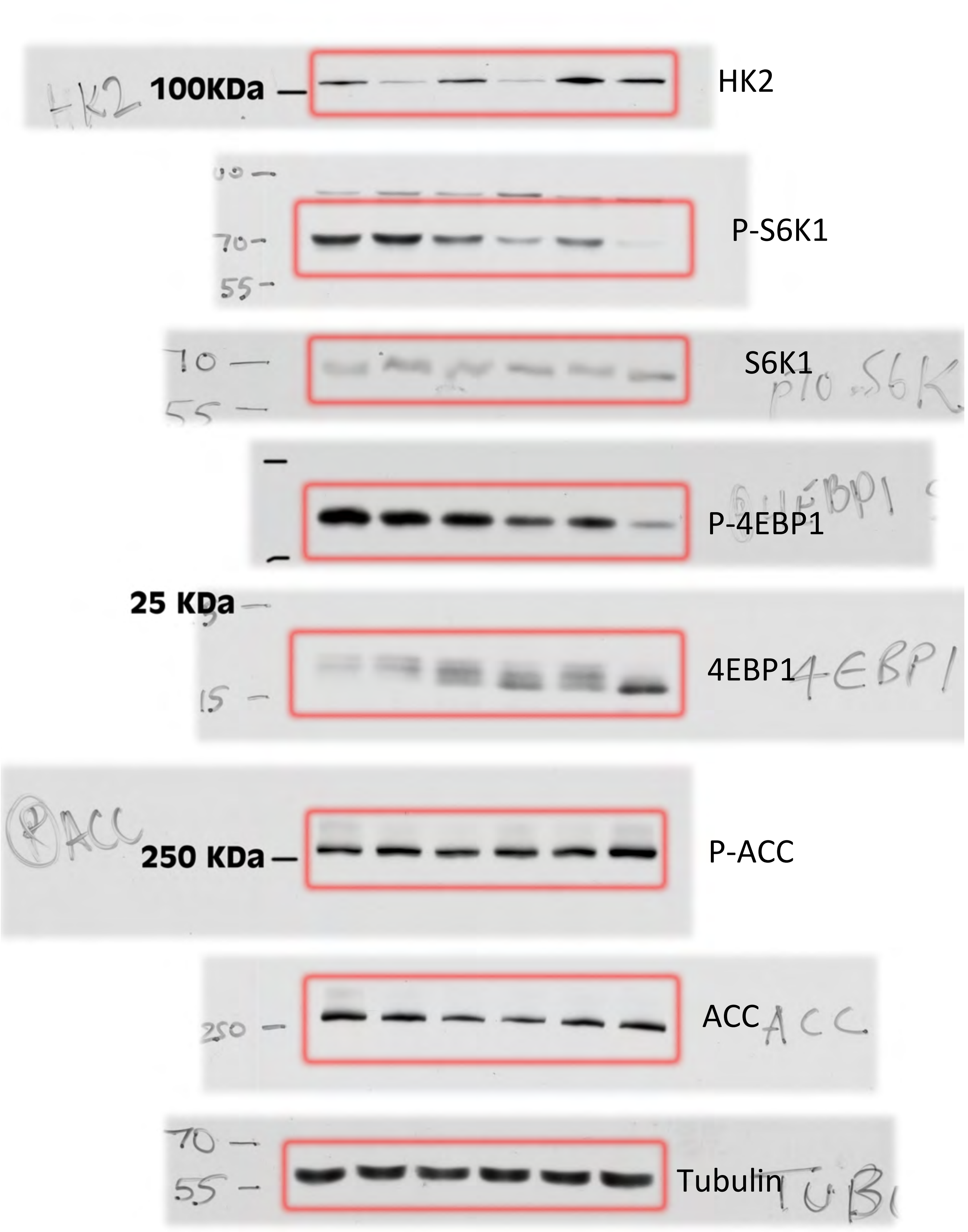

Supplementary Figure 9. Uncropped Immunoblot images

Figure 6B

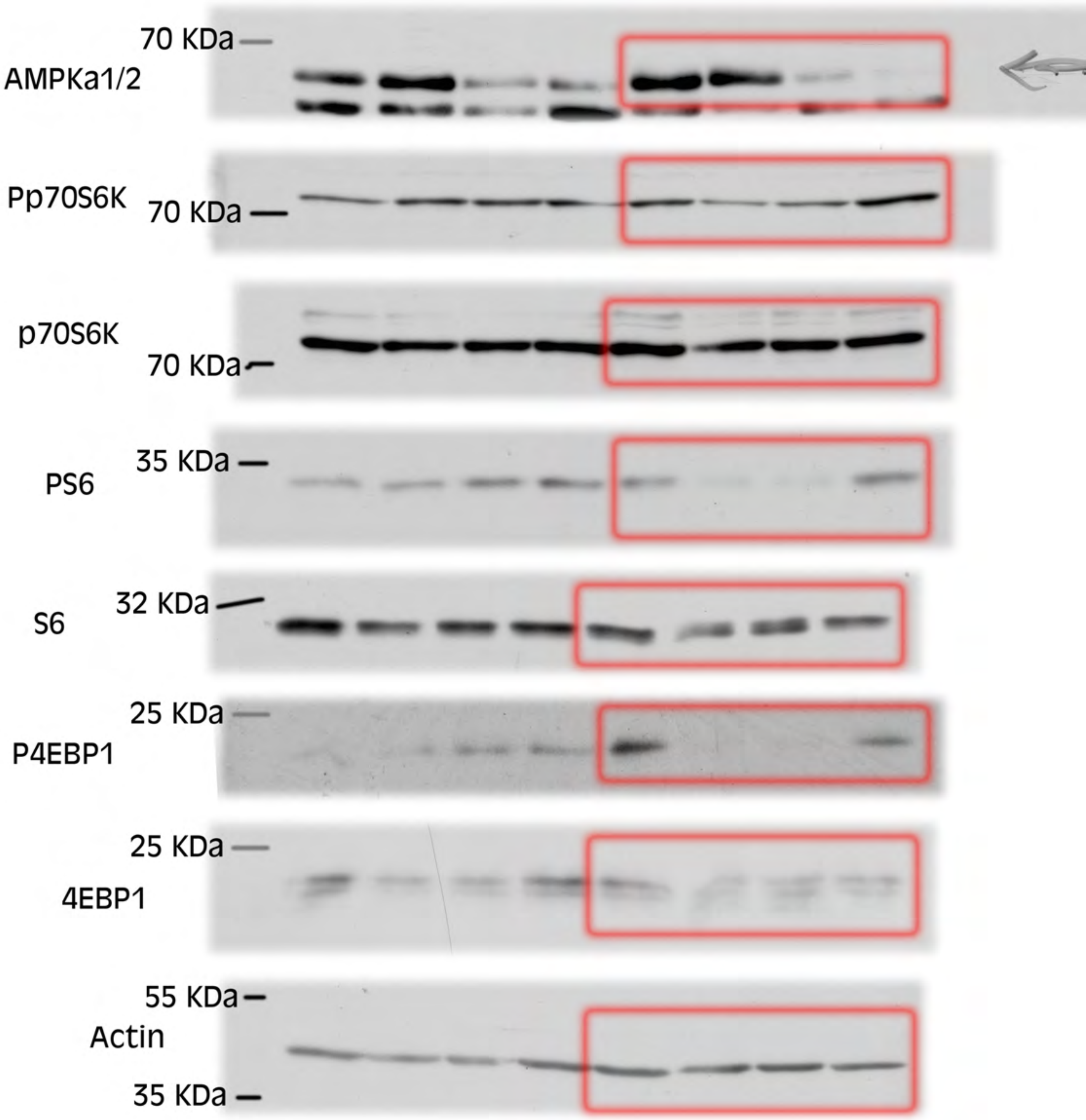

Supplementary Figure 9. Uncropped Immunoblot images

Figure 6c

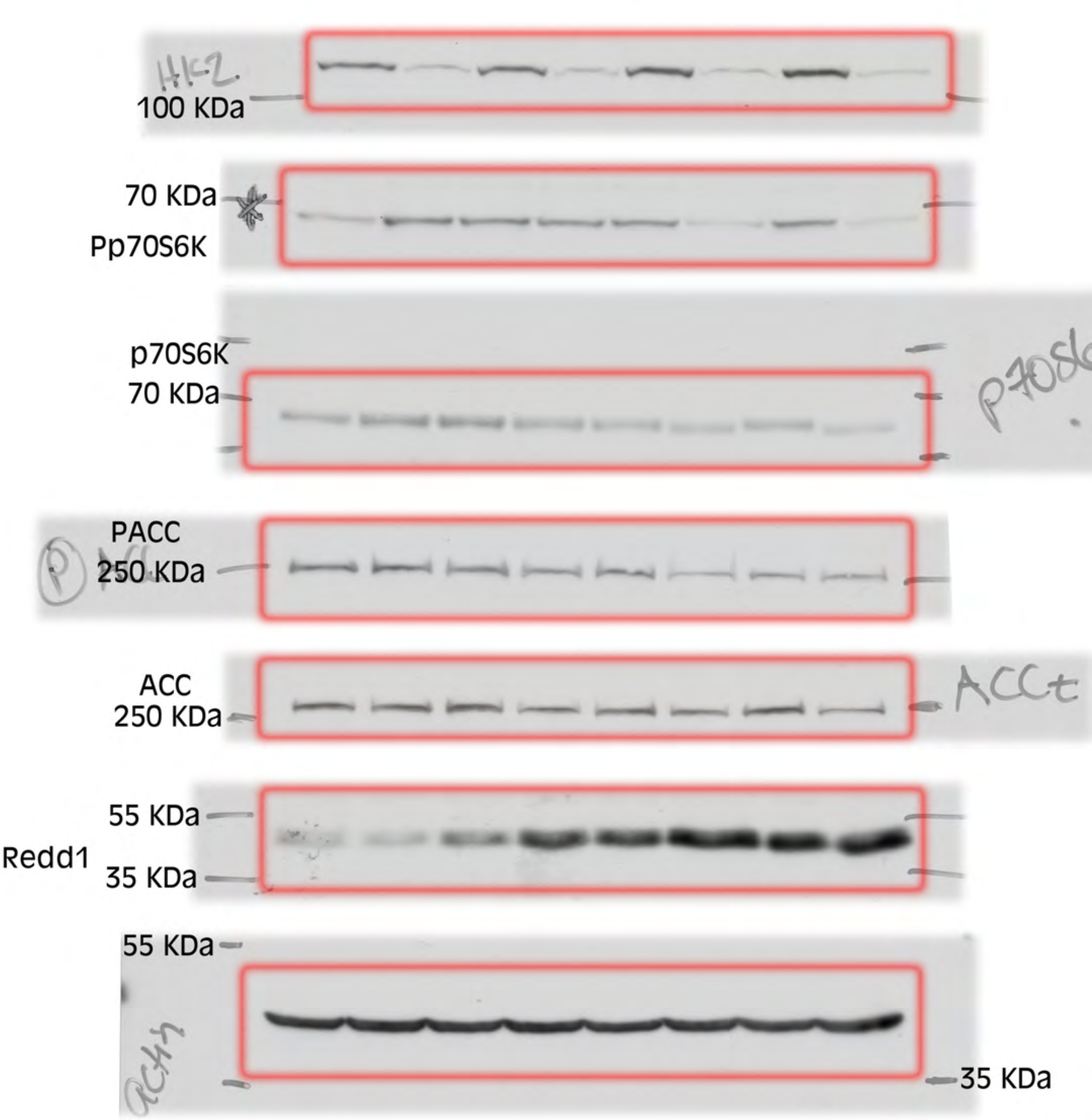

Supplementary Figure 9. Uncropped Immunoblot images

Figure 6d

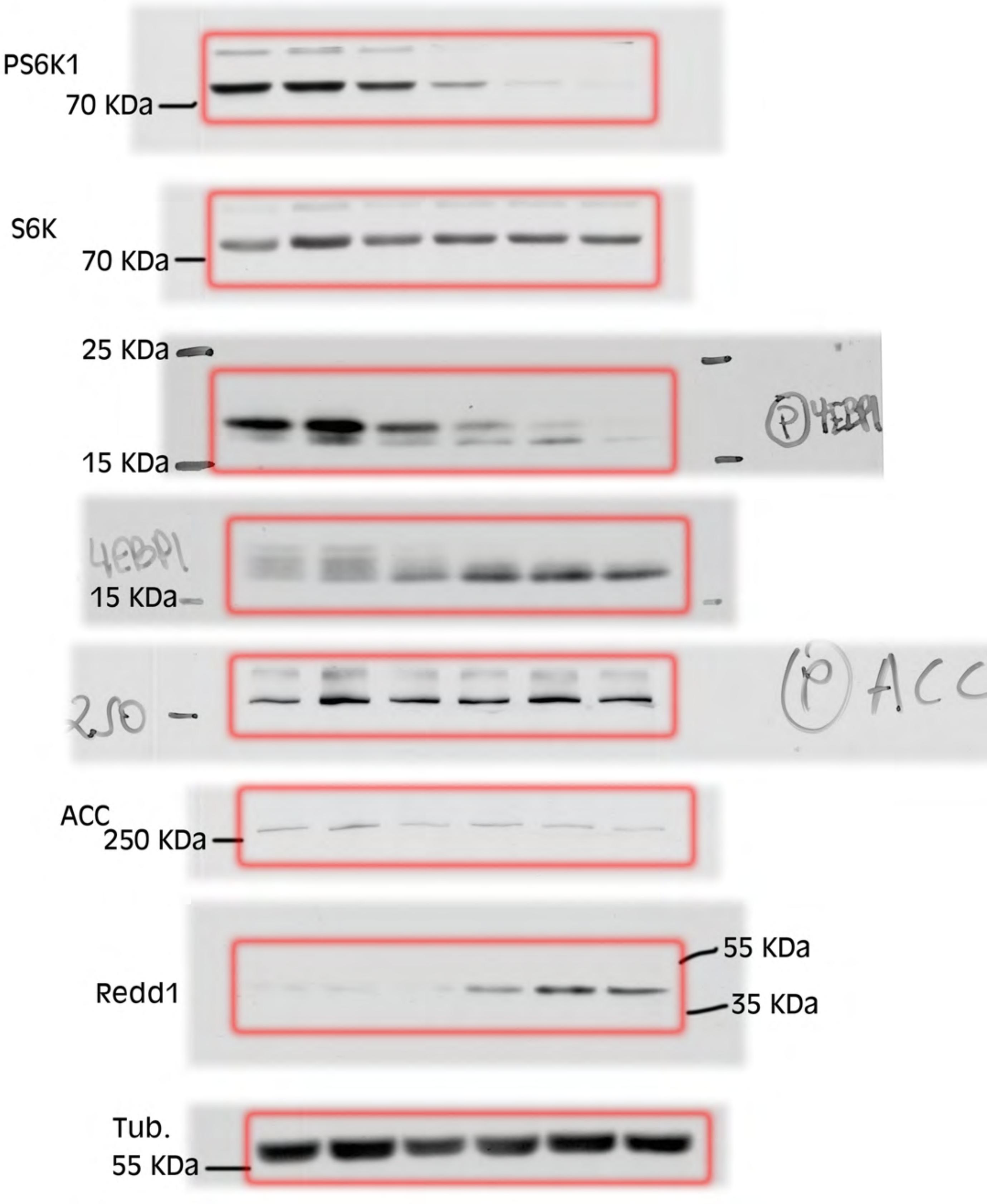

Supplementary Figure 9. Uncropped Immunoblot images

Figure 6e

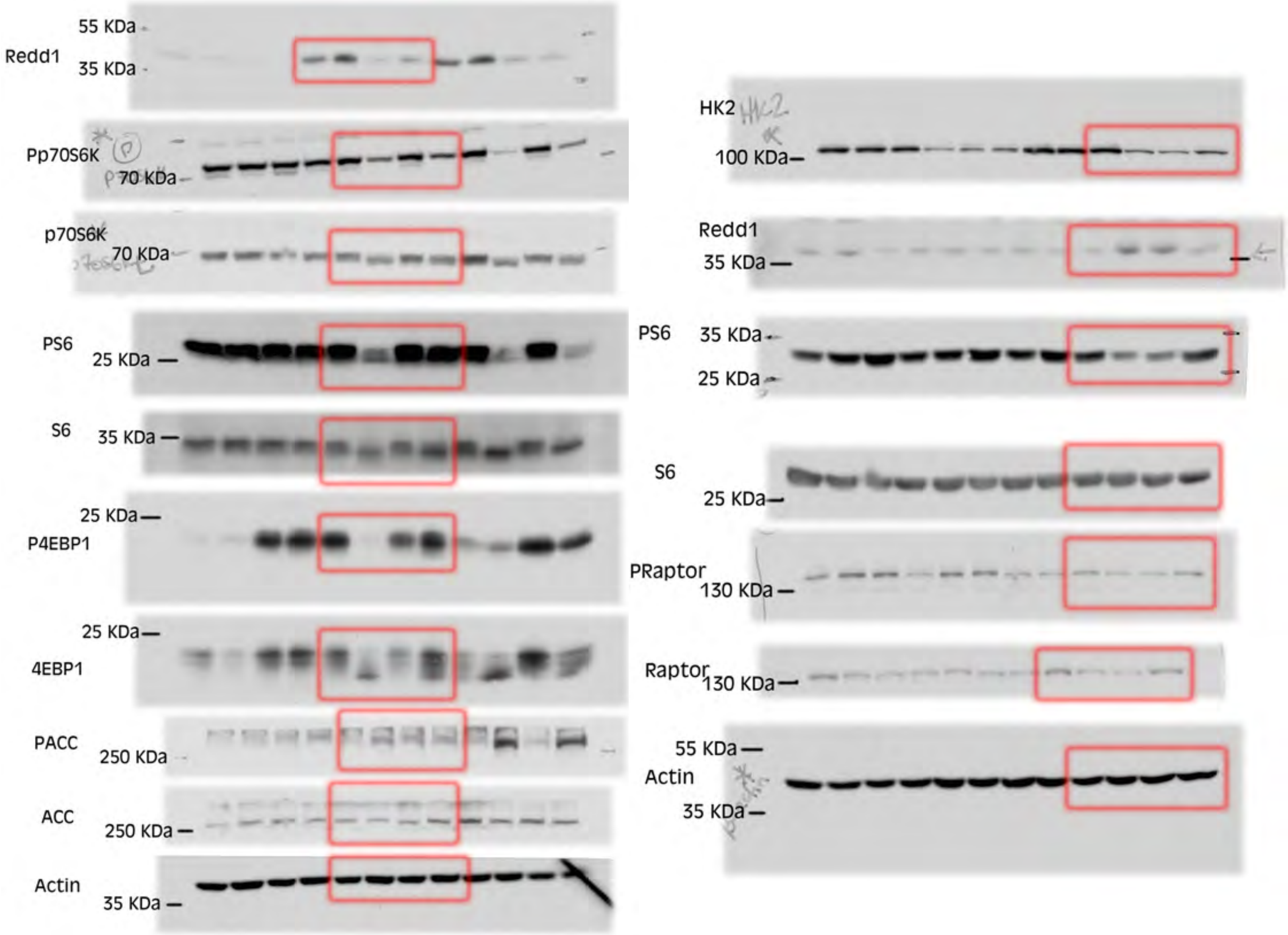

Supplementary Figure 9. Uncropped Immunoblot images

Supplementary Figure 1

HK2

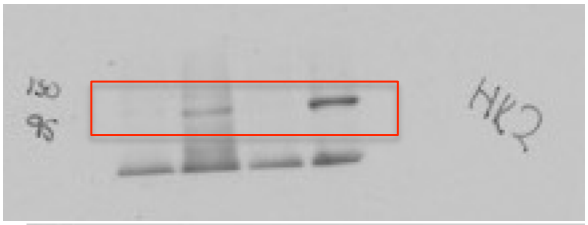

GCK

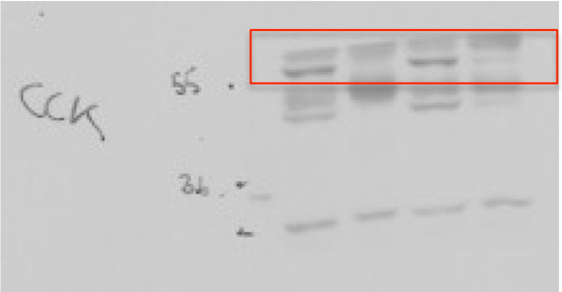

GAPDH

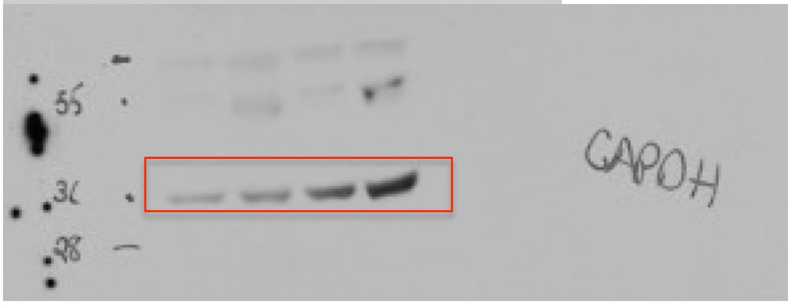

HK2

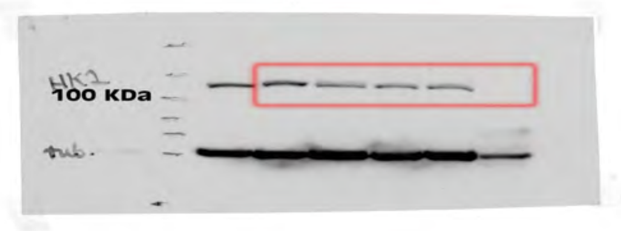

Tubulin

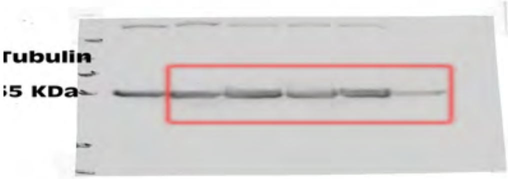

HK1/GCK

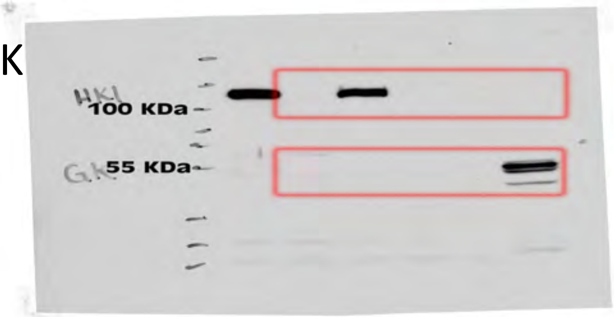

Supplementary Figure 9. Uncropped Immunoblot images

Supplementary Figure 2

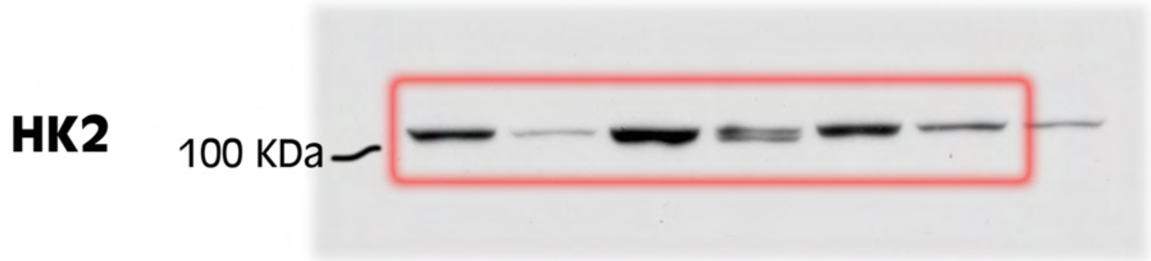

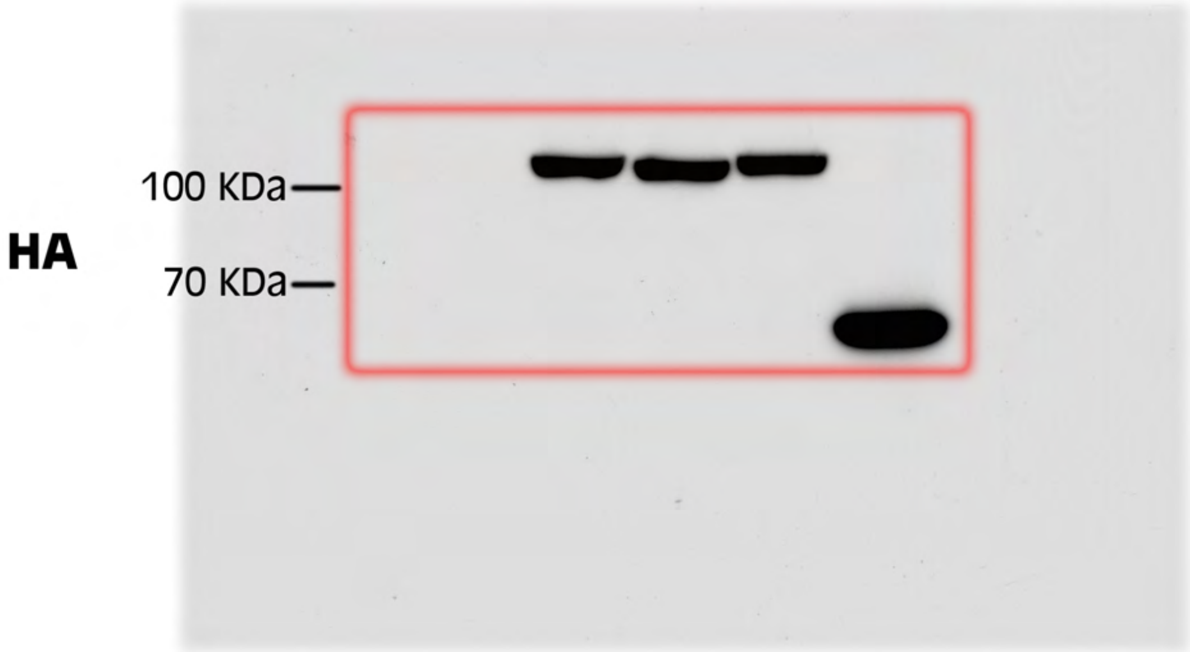

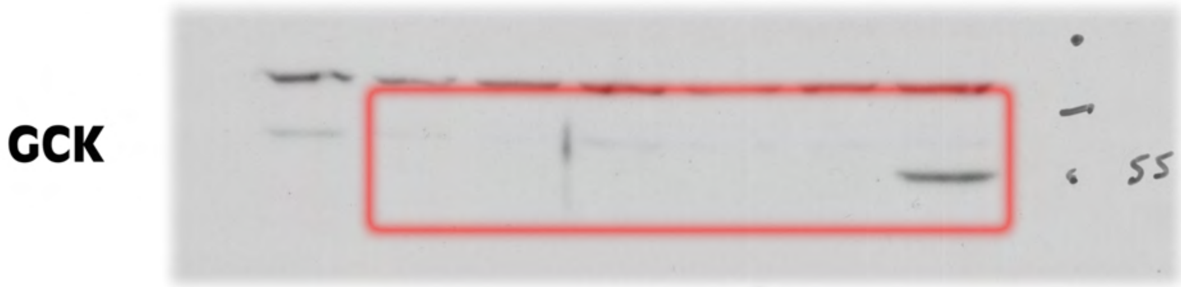

Supp. Table 1. Biomass specific uptake and production rates of extracellular metabolites (nmol/10^6cells/h, mean ± SEM, *n*=8).

	Nt-Dox	Nt+Dox	HK2-Dox	HK2+Dox
Glucose uptake	418 ± 22	474 ± 16	430 ± 15	266 ± 13
Lactate secretion	737 ± 51	845 ± 40	755 ± 25	451 ± 38
Glutamine uptake	120 ± 9	114 ± 6	125 ± 9	124 ± 4
Pyruvate secretion	50 ± 4	59 ± 3	53 ± 4	35 ± 3
Alanine secretion	26 ± 2	28 ± 4	31 ± 5	23 ± 6
Glutamate secretion	9 ± 2	10 ± 2	10 ± 2	13 ± 2
Leucine uptake	13 ± 1	13 ± 2	15 ± 3	12 ± 2
Isoleucine uptake	10 ± 1	11 ± 1	12 ± 2	11 ± 2
Valine uptake	6 ± 1	6 ± 1	7 ± 2	6 ± 1
Serine uptake	4 ± 1	6 ± 1	4 ± 1	10 ± 1

Supp. Table 2: Metabolic network model for ^{13}C metabolic flux analysis

Glycolysis			
v1	Gluc.ext (abcdef)	→	G6P (abcdef)
v2	G6P (abcdef)	↔	F6P (abcdef)
v3	F6P (abcdef)	↔	FBP (abcdef)
v4	FBP (abcdef)	↔	DHAP (cba) + GAP (def)
v5	DHAP (abc)	↔	GAP (abc)
v6	GAP (abc)	↔	3PG (abc)
v7	3PG (abc)	↔	PEP (abc)
v8	PEP (abc)	→	Pyr.c (abc)
Pentose Phosphate Pathway			
v9	G6P (abcdef)	→	Ru5P (bcdef) + CO2 (a)
v10	Ru5P (abcde)	↔	X5P (abcde)
v11	Ru5P (abcde)	↔	R5P (abcde)
v12	X5P (abcde)	↔	EC2 (ab) + GAP (cde)
v13	F6P (abcdef)	↔	EC2 (ab) + E4P (cdef)
v14	S7P (abcdefg)	↔	EC2 (ab) + R5P (cdefg)
v15	F6P (abcdef)	↔	EC3 (abc) + GAP (def)
v16	S7P (abcdefg)	↔	EC3 (abc) + E4P (defg)
Pyruvate Metabolism			
v17	Pyr.c (abc)	↔	Lact (abc)
v18	Pyr.c (abc)	→	Pyr.m (abc)
v19	Pyr.m (abc)	→	AcCoA.m (bc) + CO2 (a)
TCA Cycle			
v20	AcCoA.m (ab) + OAC.m (cdef)	→	Cit.m (fedbac)
v21	Cit.m (abcdef)	↔	AKG.m (abcde) + CO2 (f)
v22	½ AKG.m (abcde) + ½ AKG.m (fghij)	→	½ Suc.m (bcde) + ½ Suc.m (jihg) + ½ CO2 (a) + ½ CO2 (f)
v23	½ Suc.m (abcd) + ½ Suc.m (efgh)	↔	½ Fum.m (abcd) + ½ Fum.m (hgfe)
v24	½ Fum.m (abcd) + ½ Fum.m (efgh)	↔	½ Mal.m (abcd) + ½ Mal.m (hgfe)
v25	Mal.m (abcd)	↔	OAC.m (abcd)
Anaplerosis and cataplerosis			
v26	Mal.m (abcd)	→	Pyr.m (abc) + CO2 (d)

v27	Mal.c (abcd)	→	Pyr.c (abc) + CO2 (d)
v28	Pyr.m (abc) + CO2 (d)	→	OAC.m (abcd)
v29	Mal.c (abcd)	↔	Mal.m (abcd)
v30	Mal.c (abcd)	↔	OAC.c (abcd)

Fatty Acid Metabolism

v31	Cit.m (abcdef)	→	Cit.c (abcdef)
v32	AKG.c (abcde) + CO2 (f)	→	Cit.c (abcdef)
v33	Cit.c (abcdef)	→	AcCoA.c (ab) + OAC.c (cdef)
v34	AcCoA.c (ab)	→	FA (ab)
v35	DHAP (abc)	↔	Glyc3P (abc)

Amino Acid Metabolism

v36	Gln.c (abcde)	↔	Glu.c (abcde)
v37	Glu.c (abcde)	↔	Glu.m (abcde)
v38	AKG.c (abcde)	↔	Glu.c (abcde)
v39	AKG.m (abcde)	↔	Glu.m (abcde)
v40	Asp.c (abcd)	↔	OAC.c (abcd)
v41	Asp.m (abcd)	↔	OAC.m (abcd)
v42	Pyr.c (abc)	↔	Ala.c (abc)
v43	Pyr.m (abc)	↔	Ala.m (abc)

Extracellular transport

v44	Gln.ext (abcde)	→	Gln (abcde)
v45	Lact (abc)	→	Lact.ext (abc)
v46	Glu.c (abcde)	→	Glu.ext (abcde)
v47	Ala.c (abc)	→	Ala.ext (abc)
v48	CO2 (a)	→	CO2.ext (a)

Dilution from unlabeled sources

v49	AcCoA.unlabeled (ab)	→	AcCoA.m (ab)
v50	AcCoA.unlabeled (ab)	→	AcCoA.c (ab)
v51	FA.unlabeled (ab)	→	FA.c (ab)
v52	Glu.unlabeled (abcde) + Glu.c (fghij)	→	Glu.c (abcde) + Glu.snk (fghij)
v53	Asp.unlabeled (abcd) + Asp.c (efgh)	→	Asp.c (abcd) + Asp.snk (efgh)
v54	Pyr.unlabeled (abc) + Pyr.c (def)	→	Pyr.c (abc) + Pyr.snk (def)

v55	CO2.unlabeled (a) + CO2 (b)	→	CO2 (a) + CO2.snk (b)

Compartment mixing reactions

v56	Glu.c (abcde)	→	Glu.measured (abcde)
v57	Glu.m (abcde)	→	Glu.measured (abcde)
v58	Asp.c (abcd)	→	Asp.measured (abcd)
v59	Asp.m (abcd)	→	Asp.measured (abcd)
v60	Cit.c (abcdef)	→	Cit.measured (abcdef)
v61	Cit.m (abcdef)	→	Cit.measured (abcdef)
v62	Mal.c (abcd)	→	Mal.measured (abcd)
v63	Mal.m (abcd)	→	Mal.measured (abcd)
v64	Pyr.c (abc)	→	Pyr.measured (abc)
v65	Pyr.m (abc)	→	Pyr.measured (abc)
v66	Ala.c (abc)	→	Ala.measured (abc)
v67	Ala.m (abc)	→	Ala.measured (abc)

Cell growth (lumped growth reaction)

v68	0.0624 Ala + 0.0392 Arg +<br>0.0374 Asp + 0.0300 Asn +<br>0.0151 Cys + 0.0335 Glu +<br>0.0402 Gln + 0.0560 Gly +<br>0.0149 His + 0.0337 Ile +<br>0.0587 Leu + 0.0593 Lys +<br>0.0144 Met + 0.0228 Phe +<br>0.0326 Pro + 0.0447 Ser +<br>0.0402 Thr + 0.0046 Trp +<br>0.0189 Tyr + 0.0433 Val +<br>0.0290 G6P + 0.0242 Ru5P +<br>0.2335 FA + 0.0113 Glyc3P	→	Biomass
