# Supplementary material for: Hexokinase-2 depletion inhibits glycolysis and induces oxidative phosphorylation in hepatocellular carcinoma and sensitizes to metformin
Source: Nat Commun. 2018 Jan 31;9:446. doi: 10.1038/s41467-017-02733-4 (PMC5792493; doi:10.1038/s41467-017-02733-4)
Supplement: Supplementary file 2 — Description of Additional Supplementary Files [file 41467_2017_2733_MOESM2_ESM.pdf]

Description of Additional Supplementary Files

File Name: Supplementary Data 1
Description: Results of 13C-MFA
